# Multicompartment Depletion Factors for Water Consumption on a Global Scale

**DOI:** 10.1021/acs.est.2c04803

**Published:** 2023-02-28

**Authors:** Eleonore Pierrat, Martin Dorber, Inge de Graaf, Alexis Laurent, Michael Z. Hauschild, Martin Rygaard, Valerio Barbarossa

**Affiliations:** † Section for Quantitative Sustainability Assessment, Department of Environmental and Resource Engineering, Technical University of Denmark (DTU), 2800 Kongens Lyngby, Denmark; ‡ Industrial Ecology Programme, Department of Energy and Process Engineering, Norwegian University of Science and Technology (NTNU), Høgskoleringen 5, 7034 Trondheim, Norway; § Water Systems and Global Change Group, Wageningen University & Research, 6700 Wageningen, The Netherlands; ∥ Water Technology and Processes, Department of Environmental and Resource Engineering, Technical University of Denmark, ningstorvet 1152800 Kgs. Lyngby, Denmark; ⊥ Institute of Environmental Sciences (CML), Leiden University, 2300 Leiden, The Netherlands; # PBL Netherlands Environmental Assessment Agency, 2500 The Hague, The Netherlands

**Keywords:** ecosystem, freshwater availability, water management, impact assessment, sustainability

## Abstract

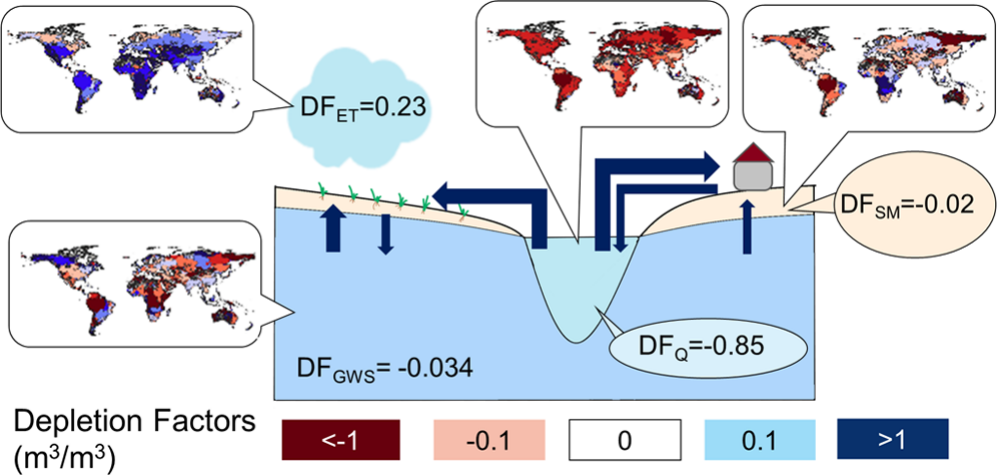

Balancing human communities’ and ecosystems’ need for freshwater is one of the major challenges of the 21^st^ century as population growth and improved living conditions put increasing pressure on freshwater resources. While frameworks to assess the environmental impacts of freshwater consumption have been proposed at the regional scale, an operational method to evaluate the consequences of consumption on different compartments of the water system and account for their interdependence is missing at the global scale. Here, we develop depletion factors that simultaneously quantify the effects of water consumption on streamflow, groundwater storage, soil moisture, and evapotranspiration globally. We estimate freshwater availability and water consumption using the output of a global-scale surface water–groundwater model for the period 1960–2000. The resulting depletion factors are provided for 8,664 river basins, representing 93% of the landmass with significant water consumption, i.e., excluding Greenland, Antarctica, deserts, and permanently frozen areas. Our findings show that water consumption leads to the largest water loss in rivers, followed by aquifers and soil, while simultaneously increasing evapotranspiration. Depletion factors vary regionally with ranges of up to four orders of magnitude depending on the annual consumption level, the type of water used, aridity, and water transfers between compartments. Our depletion factors provide valuable insights into the intertwined effects of surface and groundwater consumption on several hydrological variables over a specified period. The developed depletion factors can be integrated into sustainability assessment tools to quantify the ecological impacts of water consumption and help guide sustainable water management strategies, while accounting for the performance limitations of the underlying model.

## Abstract 2

Balancing pressures on freshwater resources requires understanding how water consumption changes water availability to humans and ecosystems. We develop regionalized depletion factors quantifying historical freshwater depletion per unit consumption volume for four key compartments of the water cycle.

## 1 Introduction

Currently, half of the global population lives in water-scarce areas, and this number is likely to increase by 2050.^1^ On the one hand, humans depend on freshwater for industrial, domestic, and agricultural uses. On the other hand, human well-being also relies on healthy terrestrial and freshwater ecosystems and ecosystem services.^2^ In many areas, human activities already extract freshwater at levels that put affected ecosystems at risk, and global water demand for all uses is predicted to increase by up to 30% by 2050.^3−5^ Flow alteration, e.g., by dam construction and water consumption, is one persistent threat to aquatic biodiversity.^6^ Water consumption has also been linked to the loss of terrestrial species, e.g., terrestrial mammals, birds, amphibians, and plants.^7,8^ A sustainable management of water resources is required, calling for a balance between anthropogenic water consumption and water availability to sustain human development while safeguarding ecosystems.^9^

New integrated approaches and tools are needed to address the challenges posed by multiple, and often conflicting, water needs for humans and ecosystems.^10^ Several tools and methods have already been proposed to tackle these issues, including water footprinting,^9,11^ planetary boundaries,^12^ integrated water resource management,^13^ life cycle assessment (LCA),^14−16^ and environmentally-extended multi-regional input–output analysis.^17^ The integrated nature of hydrological systems requires that the assessment of environmental impacts of water consumption differentiates between water compartments to reflect distributions and renewability levels among water sources.^18^ Different compartments interact with varying strengths and over a wide range of geographical and temporal scales with other components of the Earth system, such as the atmosphere, biosphere, and lithosphere. Evaluating the ecological impacts of water management decisions, therefore, requires accounting for the hydrologic processes that determine the relationships between surface and subsurface waters, as surface water, soil water, and groundwater influence one another.^19^ Existing life cycle impact assessment (LCIA) models for freshwater consumption characterize the associated damages to ecosystems and human health.^8,14,20−24^ However, the interlinkages across water compartments are rarely considered, except for a few studies modeling the recycling and transfer of evapotranspiration and LCIA models quantifying potential impacts on ecosystems.^8,21,25,26^ Several of these models are not harmonized, and their geographical scope are limited (e.g., 30% of the global wetlands or the Netherlands).^8,27^ Moreover, global LCIA models used to quantify the impacts of water consumption on freshwater ecosystems do not account for the exchanges between surface water and groundwater.^22−24^ This means that 1 m^3^ of water consumed upstream in a river basin corresponds to a reduction of 1 m^3^ downstream. In reality, river basins respond differently to water withdrawals depending on climatic and morphological conditions (i.e., connectedness of the water compartments). This needs to be modeled in an integrated way to account for the interactions between the various compartments^18^ thus allowing for differentiation of the impacts of consuming water on different ecosystems (e.g., wetlands, lakes, rivers). A framework for such a model has been proposed by Núñez et al.,^18^ but it has not been operationalized yet.

In this study, we provide a first multicompartment framework for water depletion in LCIAWe do so by quantifying the consequences of blue water consumption (i.e., from surface water and groundwater) on freshwater availability across multiple compartments and geographical regions at a global scale. To this end, we (i) describe relevant hydrological compartments and variables for human activities and ecosystem functioning and (ii) quantify the changes in these hydrological variables due to blue water consumption and the exchanges between water compartments with regionalized depletion factors. We define depletion factors for four compartments, i.e., atmosphere, groundwater, surface water, and soil, which are identified as essential to maintain the life support, climate regulation, and water storage functions of water in the global earth system.^28−30^ To satisfy the need for spatial differentiation, global coverage, and multicompartment resolution, we rely on a physically-based surface water–groundwater hydrological model running at a high resolution (i.e., 5 arc min, ∼10 km × 10 km at the equator), globally.

## 2 Materials and Methods

### 2.1 Modeling Scope

**Figure 1:**
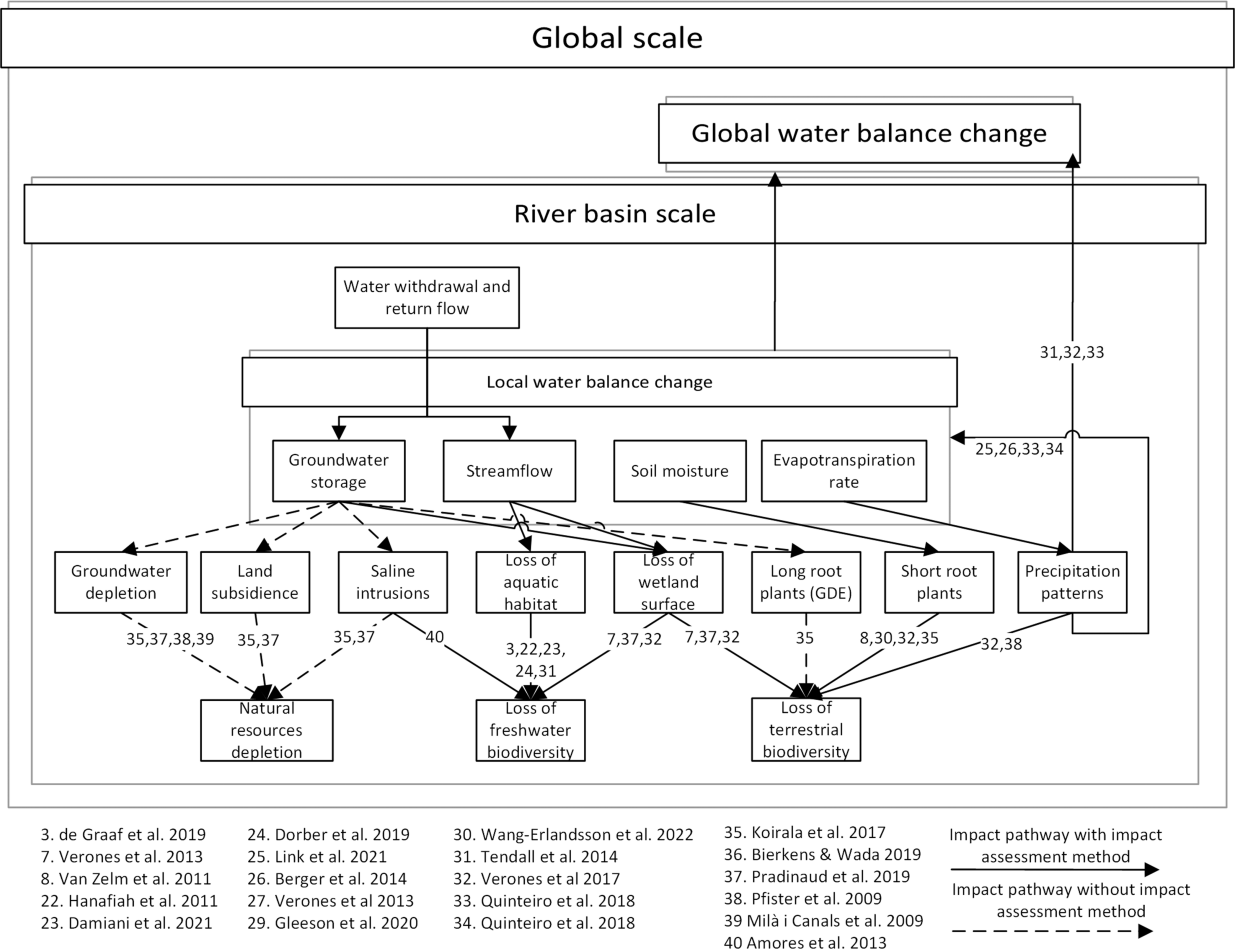
Cause-effect chain linking water consumption to hydrological indicators and subsequently to ecosystems and freshwater natural resources.

Water availability in the surface water, groundwater, soil, and atmosphere compartments can be represented by several different hydrological variables. In this study, we selected key hydrological variables for ecosystem functioning and human livelihoods based on their environmental relevance, i.e., streamflow (Q), groundwater storage (GWS), evapotranspiration rate (ET), and soil moisture (SM) (Table 1 and Figure 1) by reviewing the literature and published life cycle impact assessment methods. We investigate how surface and groundwater water consumption (i.e., blue water) influence the hydrological variables by calculating hydrological indicators (*D*_i_ for i equal to Q, GWS, ET, SM) defined as the cumulated change over time in the variables induced by blue water consumption (Table 1). Streamflow change is potentially detrimental to wetland and freshwater biodiversity because it directly affects freshwater habitat size and suitability.^7,22−24,27,31,32^ Soil moisture and evapotranspiration are key to the thriving of vegetation and the coupling between terrestrial water compartments and precipitation.^29,30^ Evapotranspiration changes alter air moisture and regional precipitation regimes, thus they possibly damage ecosystems by reducing green water for natural vegetation and crops as well as blue water for freshwater ecosystems and human supply.^25,26,30,33,34^ Soil drying affects vegetation activity and can potentially lead to species extinctions.^8,30,35^ Groundwater storage and streamflow are equally relevant to human water supply, as ∼52.0 and 47.7% of total global water withdrawals come from groundwater and surface water (the remaining 0.3% is desalinated).^5^ Groundwater storage change can lead to saline intrusions, groundwater depletion, and land subsidence that reduce the availability of groundwater to humans.^36−39^ It can also damage freshwater and terrestrial biodiversity. Groundwater storage and discharge support river baseflow,^3^ groundwater-dependent ecosystems,^8,35^ and groundwater-fed wetlands,^7^ while saline intrusions can affect coastal streams and wetlands.^40^ Therefore, changes in the hydrological variables streamflow, soil moisture, evapotranspiration, and groundwater storage, in particular freshwater loss, can put at risk the integrity of ecosystems and human communities.

**Table 1:** Selected Hydrological Indicators for Estimating Depletion Factors.

compartment	hydrological variable	unit of the variable	description of hydrological indicator
surface water	streamflow Q(*t*)	m^3^·year^–1^	Change of streamflow, i.e., river discharge, at the outlet of the river basin *D*_Q_(*t*) expressed in (m^3^·year^–1^). For each year (*t*) between 1960 and 1990, 10 years moving averages were calculated.
groundwater	groundwater storage GWS(*t*)	m^3^	Change of groundwater storage volume *D*_GWS_, in both confined and unconfined aquifers, between 1960 and 2000 (expressed in m^3^). The volume change is estimated based on simulated groundwater head drawdown and aquifer storativity and specific yields (i.e., the volume of groundwater released from a unit area of aquifer for a unit drawdown of groundwater head). The groundwater head drawdown is the difference between the annual average groundwater head in the decades 1990–2000 and 1960–1970.
soil	soil moisture SM(*t*)	m^3^	Change of soil moisture volume *D*_SM_ over the top 1.5 m of soil depth between 1960 and 2000 (expressed in m^3^). The change is the difference between the annual average soil moisture in the decades 1990–2000 and 1960–1970.
atmosphere	evapotranspiration rate ET(*t*)	m^3^·year^–1^	Change of evapotranspiration rate from vegetation, bare soil, and open water *D*_ET_ (expressed in m^3^·year^–1^). For each year (*t*) between 1960 and 1990, 10 years moving averages were calculated.

In hydrogeology, the term groundwater depletion refers to the persistent loss of groundwater volume and decline of groundwater levels, resulting from the long-term withdrawals from the aquifer at a rate exceeding the annual groundwater recharge.^36^ Groundwater depletion also increases the aquifer capture, i.e., the reduction of aquifer discharge or the increase of recharge, thus possibly resulting in streamflow depletion and loss of evapotranspiration.^3,36,41^ Different from scarcity, which represents the competition between humans and ecosystems for available freshwater resources on a yearly (or monthly) basis, depletion is the multiannual (e.g., 40 years) loss of freshwater in a given region induced by water consumption.^42^ In this study, we extend the concept of water depletion to the soil, surface water, and atmosphere compartments, introducing the hydrological indicators *D*_i_ for each hydrological variable *i* (noted *D*_Q_, *D*_GWS_, *D*_SM_, *D*_ET_ and defined in Table 1, Section 2.3.1, and Table S2 provides extended calculation details) quantifying the change of the hydrological variables induced by total blue water consumption. The hydrological indicators are calculated for a 40-years historical period (1960–2000) so that they reflect long-term ongoing water transfer processes. *D*_i_ describes average trends that are useful to model potential environmental impacts in LCIA. The year 1960 was deemed an acceptable reference state because water consumption rates have been increasing since the 1950s when irrigated agriculture started to expand globally. Note that the absolute value of the groundwater storage GWS(*t*) is unknown and only the groundwater storage change *D*_GWS_ is quantified (Table 1).^5^ It represents the total groundwater availability change, including the exchanges with rivers, soil, and the atmosphere.^36^

### 2.2 Global-Scale Surface Water–Groundwater Model

We used the physically based global-scale surface water–groundwater model PCR-GLOBWB-MF, simulating groundwater and surface water hydrology at high resolution and including water demand and water use from three different sectors, i.e., the domestic sector, the industry, and agriculture (i.e., irrigation and livestock).^3^ Hereafter, the model is called GSGM, its features and performance are comprehensively documented in the literature.^3,43−46^ The GSGM performs a dynamic simulation of water consumption and groundwater–surface water interactions. The dynamic modeling of these interactions is a unique feature of the model and a prerequisite for analyzing the effects of groundwater withdrawal on streamflow (Figure S7). The groundwater model Modflow (MF) simulates groundwater heads and groundwater flows in the aquifer in 3D. While the lateral groundwater flows can contribute significantly to the water budget of river basins, the groundwater head governs the interactions between groundwater and soil, and groundwater and rivers.^43,47^ The hydrological model (PCR-GLOBWB, Figure S7b) and the groundwater model (MF) are fully coupled to compute the interactions between surface, groundwater, and soil. It also includes a vegetation compartment, where the land cover is considered static; therefore, it models crop water use (from precipitations and soil). The coupled model runs at 5 arc-min resolution and at daily timestep. It includes a water demand and water use module that dynamically allocates sectoral water demands from irrigated agriculture, industries, households, or livestock to withdrawal of desalinated water, groundwater, or surface water based on the availability of these resources (Figure S7c).^45^ Moreover, surface water withdrawals are limited by an environmental flow requirement, as legislation usually prescribes a minimum streamflow.^45^ Return flows of unconsumed withdrawn water, flowing back to groundwater or surface water resources, are included in the estimate of water availability and are sector-specific. The strength of the dynamic allocation scheme is that it does not depend on data on groundwater withdrawal fractions for a specific year or region. Thus, the GSGM is more flexible when simulating the global hydrological system over a long period in the context of climate change.^45^Section S1.1 provides details of the GSGM.

Published results from the GSGM provided grid-cell estimates of routed monthly surface water streamflow (q_k_(t)), monthly groundwater head (h_k_(*t*)), annual soil moisture (sum of top and bottom soil moisture storage sm1_k_(*t*) + sm2_k_(t)), and annual evapotranspiration (et_k_(t)), as well as other central model inputs, such as annual net water consumption rate (wc_k_(t)), grid cell area (a_k_), and aquifer storativity (Sy, i.e., the volume of groundwater released from a unit area of aquifers for a unit drawdown of groundwater head) (see Figure S7, calculation details in Sections S1.2 and 1.3 and Table S1).^3^ The water consumption rate is defined as the difference between withdrawals and return flows. The grid cell return flows are assumed to happen in the same grid cell as withdrawals. Yet, return flows to surface water can influence downstream surface water availability due to river routing, and return flows to groundwater may influence streamflow downstream through surface water–groundwater interactions that are explicitly included in the model.

We focus on blue water consumption consequences only; thus, we removed the influence of green water consumption and climate variations on the water balance. The model was run twice: once including manmade perturbations in the form of surface water and groundwater withdrawals, dams, and reservoirs (i.e., a human-impacted run) and once without water consumption or dams (i.e., a natural run). The human-impacted run reflects the influence of climate, land use, and blue water consumption on the hydrological cycle, while the natural run only includes the effect of climate and land use. To derive the depletion factors, we subtract the *D*_i_ calculated with the natural set from the *D*_i_ calculated with the human set to remove the influence of background hydrological processes on the *D*_i_. In doing so, we isolate the effect of blue water consumption (incl. desalination) and dams on the water system (6862 dams) and remove the effect of climate change and land use from the DF_i_.^48−50^

### 2.3 Depletion Factor Modeling

#### 2.3.1 Depletion Factors

In hydrogeology, the capture fraction and the depletion potential indicators estimate the streamflow depletion due to additional groundwater pumping over time.^41,51,52^ Similarly, we define the depletion factors in this study as the historical rate at which water availability in each compartment, represented by the selected hydrological indicators, is affected by blue water consumption (Table 1). Because the consequences of blue water consumption occur after a delay, which varies for each compartment and each river basin, we define DFs that represent the dynamic evolution of the water balance over the period 1960–2000. In a river basin, the change in storage over time is equal to the cumulated flows in and out of the boundaries of the river basin. The depletion factors (DF_i_) for each hydrological indicator *i* (noted DF_Q_, DF_GWS_, DF_SM_, DF_ET_) are derived from the equation of the water balance of a river basin over the period 1960–2000, as explained in Sections S1.4 and S1.5. Each DF_i_ corresponds to a selected term of the water balance representing the change in the compartment relative to the blue water consumption as in eqs 1 and 2.

For evapotranspiration and streamflow, depletion factors follow eq 1, which is similar to the equation for transient multimedia fate factors proposed by Núñez et al.^18^
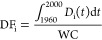
1where WC (expressed in m^3^) is the cumulated net water consumption from the river basin’s surface and groundwater from 1960 to 2000 (see eqs S1–S4 and S29). The *D*_Q_ and *D*_ET_ (expressed in m^3^·year^–1^) are integrated over time to obtain an estimate of the cumulated volume change leaving the river basin to the ocean (∫*D*_Q_(*t*)d*t*) and the atmosphere (∫*D*_ET_(*t*)d*t*) from 1960 to 2000. We corrected for climate influence by subtracting (∫*D*_Q_(*t*)d*t*) and (∫*D*_ET_(*t*)d*t*) calculated with the natural set. After this correction, the numerator of eq 1 is interpreted as the cumulated change of streamflow (*D*_Q_) and evapotranspiration (*D*_ET_) caused by blue water consumption and is expressed in (m^3^). Therefore, the hydrological indicators (*D*_i_) represent the changes induced by human blue water consumption only, excluding other influential factors, such as climate change and green water use (i.e., rainfall part of the evapotranspiration). For groundwater storage and soil moisture, the depletion factor is the ratio between the hydrological indicator *D*_i_ (*D*_GWS_ and *D*_SM_, respectively) and cumulated blue consumption volume WC following eq 2. Equation 2 includes the cumulated change of the storages GWS and SM over time following the literature about groundwater depletion.^5,36,43,48^ Cumulated values are used to avoid depletion double-counting from one year to the next.
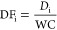
2where *D*_i_ is the difference of hydrological indicator i groundwater storage and soil moisture between 1960 and 2000 expressed in (m^3^). We also corrected the influence of climate and hydrological background on *D*_GWS_ and *D*_SM_ calculated with the human-impacted set by subtracting the cumulated changes calculated with the natural set.

The WC and the *D*_i_ at the river basin scale were derived from the GSGM data for the human and the natural sets following the procedure described in Sections S1.3–S1.5. First, the river basin-scale annual average of each hydrological indicator was computed, summing the grid cells’ values for wc_k_(*t*), gws_k_(*t*), sm_k_(*t*), et_k_(*t*) and selecting the value of the grid cell at the river mouth for q_k_(*t*) (Section S1.3). We calculated the 10-year running averages of streamflow, evapotranspiration, and soil moisture to reduce the influence of interannual variability on the depletion factors. Running averages were also applied to groundwater head and groundwater storage to harmonize the interpretation of depletion factors across hydrological indicators. Running averages were deemed acceptable because the objective of the depletion factors is to summarize large-scale anomalies and trends. Therefore, the *D*_i_ represents the cumulated change between the decades 1960–1970 and 1990–2000.

Because the compartments are hydrologically interconnected, changes in the hydrological variables *D*_i_ can be directly induced by water consumption in the same compartment or indirectly by the change in the boundary flows at the interface with other water compartments. For example, streamflow depletion can result from direct pumping in the river or the groundwater drawdown induced by groundwater pumping. Therefore, the DFs include the exchanges between compartments, and they can represent either a loss or a gain of water volume in the river basin so that the loss in one compartment in one region can be compensated by a gain in other compartments and regions. Eventually, negative DF_i_ values thus indicate a loss and positive DF_i_ values indicate a gain of water in the considered compartments between the decades 1960–1970 and 1990–2000. For example, a DF_Q_ value of −0.1 (streamflow) means that each m^3^ of water consumed has induced a reduction in the streamflow discharge volume of 0.1 m^3^ historically in the river basin. The DFs are dimensionless (m^3^ cumulative change/m^3^ cumulative consumption) and are derived from the water balance change over a specific period and river basin, which allows comparing the sensitivity of the different compartments to blue water consumption.

#### 2.3.2 Spatial Aggregation at the Basin Scale

We modeled and reported the depletion factors at the river basin scale (calculation details in Section S1.2), here defined as the hydrologically connected portion of land with an outlet to the sea or an internal sink. We considered the river basin scale large enough to support the assumption that the change of streamflow, groundwater storage, soil moisture, and evapotranspiration within a river basin relates only to the consumption in the same river basin, i.e., consumption and return flows of surface water occur in the same basin. This is backed by the fact that human impacts on freshwater ecosystems are often studied at the river basin scale, as river basin boundaries represent impassable barriers for most aquatic species.^53^

The river basin boundaries were delineated based on the hydrologically conditioned digital elevation model used in GSGM to ensure consistency with streamflow data. This resulted in a total of 20 317 river basins with a median area of 683 km^2^ and a range of 11–5 912 646 km^2^. In basins with low consumption, i.e., below the threshold of the 25% percentile of total consumption volume over the period 1960–2000 (i.e., <0.01 mm^3^/year), we assumed that water consumption estimates in the GSGM were too uncertain to yield meaningful results.^54^ Therefore, we decided to exclude those 11 654 river basins, corresponding to 13% of the global landmass (excl. Antarctica and Greenland) and 7% of the landmass, where consumption occurs that is roughly the combined size of deserts in Australia, Africa, and Asia (Figure 1, the land surface in gray).

The GSGM outputs were aggregated at the river basin scale to calculate the DFs, following the calculation procedure described in Sections S1.2 and S1.3.

## 3 Results and Discussion

### 3.1 Global Consumption Increase and Associated Depletion

**Figure 2:**
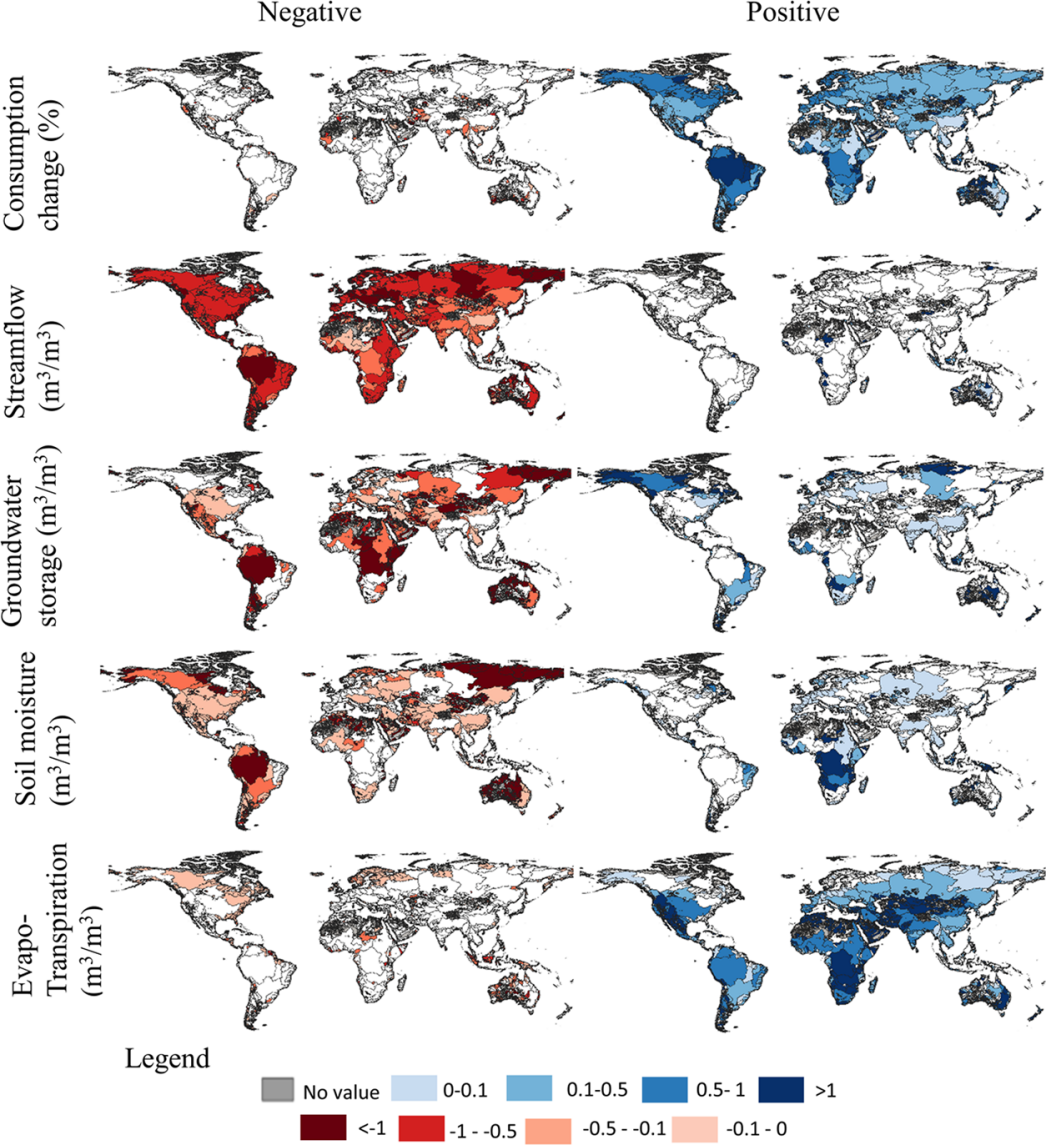
Global maps of water consumption change and resulting depletion factors for streamflow, groundwater compartment storage, soil moisture, and evapotranspiration for 8664 river basins from 1960 to 2000. The effect of water consumption on the different depletion factors is split between positive (left) and negative (right) values for simplicity of representation.

Global annual consumption of freshwater was estimated to have increased by ∼20% between 1960 and 2000 (from 2200 to 2625 km^3^·year^–1^) in the GSGM. Consumption has increased for over 90% of the analyzed landmass (Figure 2). Consequently, streamflow (Q), groundwater storage (GWS), and soil moisture (SM) have decreased, locally, as median depletion factors (DFs) were found to be negative (Figure 2 and Table 2) over more than 61% of the analyzed landmass. Water consumption increased evapotranspiration (ET) for 83% of the total landmass (negative DF) at a median rate of 0.23 m^3^/1 m^3^ water consumption. Moreover, the groundwater–surface water withdrawal ratio was on average 0.05 (area-weighted median, 25th percentile: 0.01; 75th percentile: 0.37), which means that surface water consumption is more intense than groundwater consumption over the considered period (river basins’ ratios are shown in Table 3).

**Table 2:**
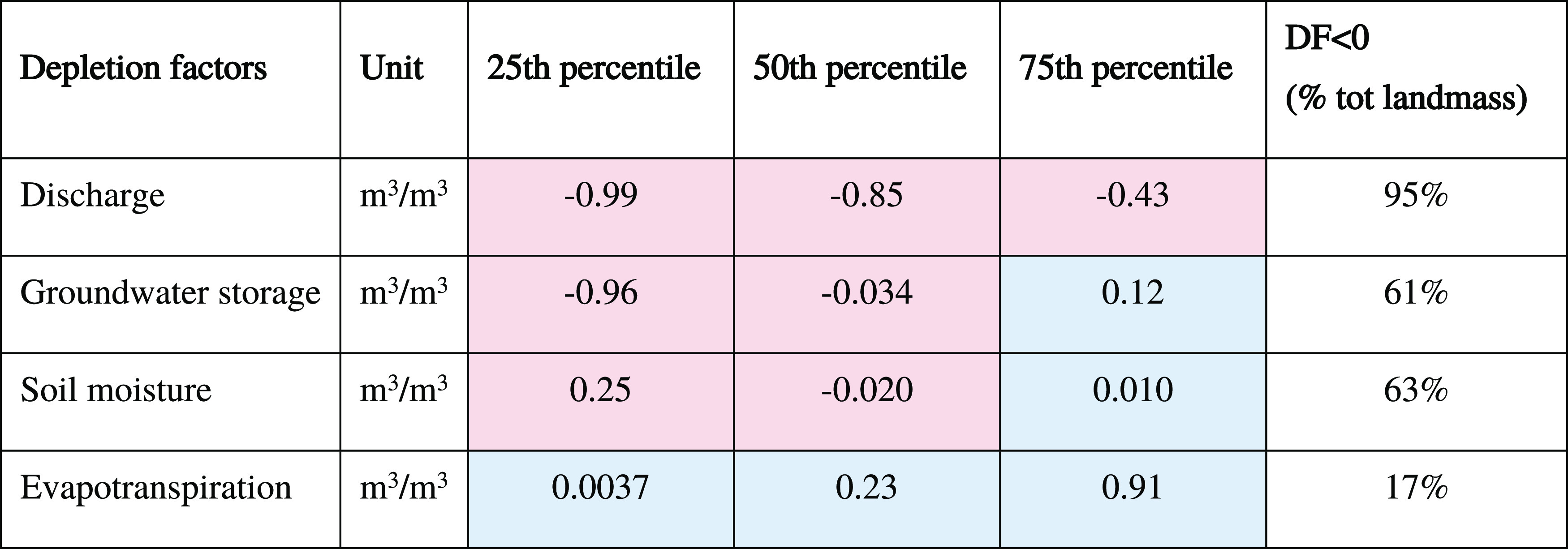
Depletion Factor Interquartile Range Results^a^

a Negative and positive values are shown in red and blue, respectively. Percentiles are calculated on the depletion factors mapping at 5 arc min spatial resolution. The total landmass excludes Greenland and Antarctica.

**Table 3:**
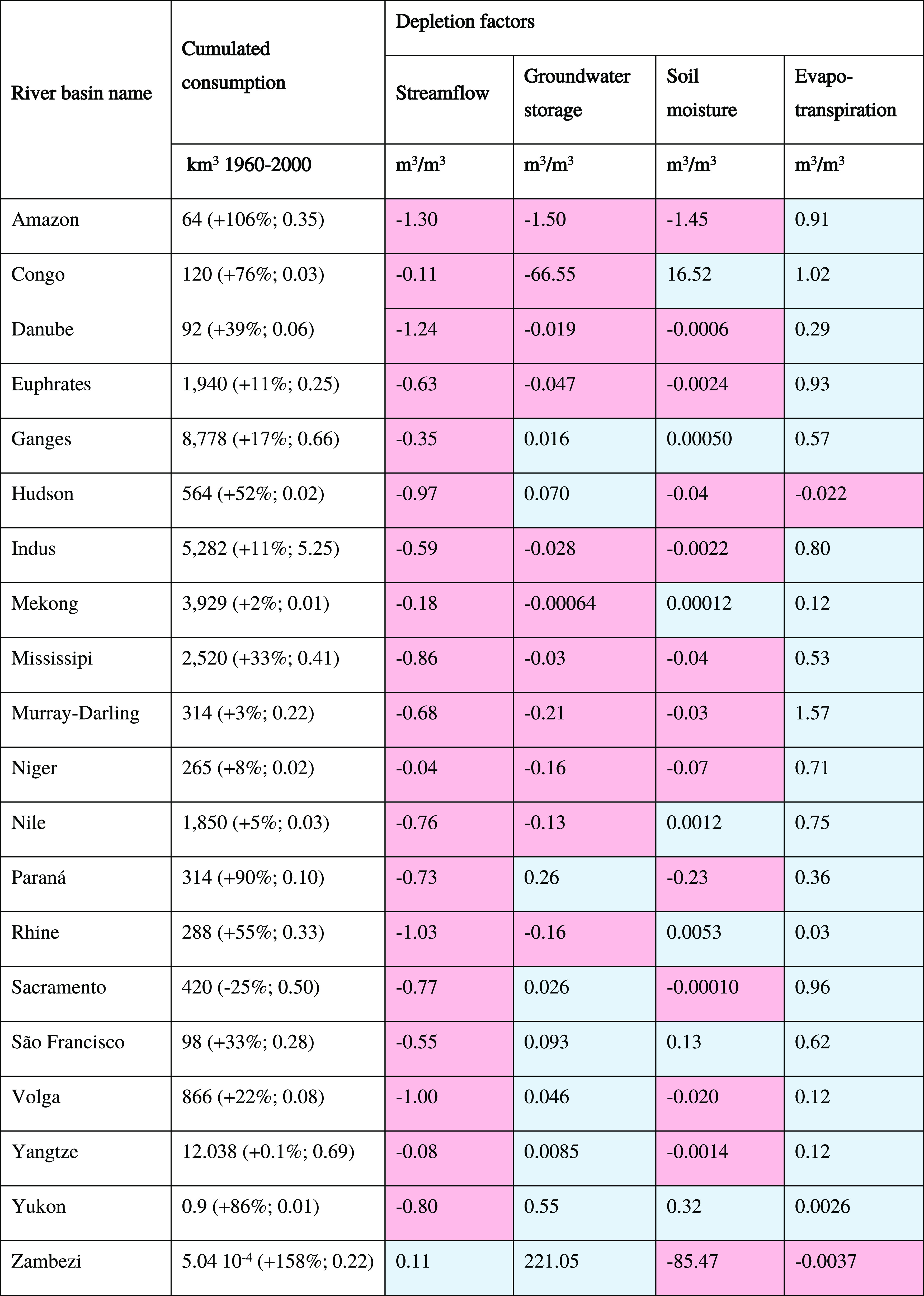
Depletion Factors and Cumulated Consumption for Major River Basins Around the World^a^

a Positive and negative depletion factors are reported in blue and red, respectively. Consumption change and average groundwater–surface water consumption ratio are reported in parenthesis after the cumulated consumption. The consumption change is relative to the mean value over the period 1960–2000.

The global situation, represented by the median DF values in Table 2, can be illustrated by the cases of the Ganges and Indus River basins (Figures S2 and S3). In the Indus River basin, negative DFs were obtained for streamflow, groundwater storage, and soil moisture (Table 3), thus confirming earlier observations and scarcity assessments that intense freshwater consumption has reduced surface and groundwater availability.^42,55^ In the Ganges River basin, only streamflow decreased (negative DF), while groundwater storage, soil moisture, and evapotranspiration increased (positive DF). Thus, the overall water depletion due to consumption is more intense in the Indus River basin than in the Ganges River basin. Positive DFs for evapotranspiration and soil moisture found in both river basins indicate that irrigation provides soil moisture to support crop growth. A positive groundwater storage DF in the Ganges River basin suggests that return flows from irrigation are recharging the aquifer. In contrast, return flows are not compensating groundwater withdrawals in the Indus River basin (i.e., negative DF). These results are consistent with the study of MacDonald et al. of the Indo-Gangetic aquifer system, where the Indus River basin and the Upper Ganges River (western part of the Ganges River basin) are reported to be subject to intense groundwater depletion, causing streamflow infiltration into the aquifer and streamflow reduction at the Indus River mouth.^55^ The authors also found that the situation in the lower Ganges River basin has not been as critical (eastern part of the Ganges River basin), with average null groundwater table drawdown, and no river infiltration to the aquifer. The depletion factors portray the average freshwater availability change at the basin scale (from 1960 to 2000), but the local differences between irrigated cropland versus non-irrigated land (farms and natural vegetation) are masked. For instance, SM decrease and ET increase dominate in the Indus River basin, but, locally, evapotranspiration may decrease for natural vegetation and non-irrigated farms. Irrigation is the most important water use in terms of consumption volume, representing from 70 to 90% of global modeled withdrawals and on average 54% of country withdrawals, according to FAO’s statistics in 2018.^5,56,57^ Irrigation substitutes insufficient soil moisture to support crops’ transpiration at optimal rates, coherently with the irrigation demand calculation scheme of the GSGM. Thus, positive evapotranspiration DFs were found in areas of marked irrigation practices (Figure 2).

Streamflow depletion has been the most widespread effect of consumption since 1960 with 7,795 out of 8,502 river basins being impacted, i.e., 95% of the analyzed landmass, due to the continuous increase in water consumption rates from 1960 to 2000 (Figures 2 and S1) and the relatively low groundwater–surface water ratio. Streamflow reduction comes from the short-term effects of direct withdrawals from rivers and the delayed, indirect effects of groundwater pumping. High surface water consumption possibly comes from the better accessibility of surface water and average lower pumping cost, as groundwater pumping cost increases with groundwater table drawdown.^58^ In the GSGM, surface water withdrawals occur before groundwater, as long as the streamflow is higher than the environmental flow requirements.^45^ Moreover, in ∼40% of the river basins where groundwater is used, increased aquifer capture contributes to streamflow decrease.^47^ In this case, water from the river would continue flowing to the aquifer even though all water consumption would stop. Therefore, streamflow depletion can occur in the long term (40 years) despite higher renewability rates compared to groundwater.

The largest median depletion factors were found for the surface water compartment (streamflow DF). Comparing the impact of consuming 1 m^3^ of water on aquifers and streamflow water availability, the area-weighted water loss in streamflow (−0.85 m^3^) is 25 times higher than that in the aquifer (−0.034 m^3^) and 43 times higher than soil moisture (−0.019 m^3^).

Global groundwater depletion was estimated to 94 km^3^/year based on the results (3800 km^3^ from 1960 to 2000; in line with de Graaf et al.^3^), which is consistent to previous estimates 113 km^3^·year^–1^ from 2000 to 2009^48^ and from ∼70 to 333 km^3^·year^–1^ from 1960 to 2010.^59,60^ High groundwater storage depletion may stem from the longer response time of aquifers, i.e., time to reach equilibrium, which depends on recharge rate and hydrogeological properties of the subsurface (e.g., transmissivity). Aquifer response time estimates range from 10 to 1000 years in regions, where groundwater consumption takes place.^61^ Based on groundwater response time maps, 2890 (33%) of the river basins with groundwater response time below 50 years have DF_Q_ and DF_GWS_, representing the dynamics of water transfers over the period 1960–2000.^61^ These basins are generally small and located, for example, in Italy, Denmark, Southern Norway, Iceland, Western Germany (Rhine basin), and Central America. Thus, groundwater storage can be considered depleted over the period in the remaining 5774 (67%) river basins, which represents most of the analyzed landmass. In these regions, streamflow depletion induced by groundwater pumping is delayed since surface–groundwater interactions occur at a larger time scale than the considered 40 years period. de Graaf et al. found that between 17 and 21% of the river basins already face streamflow depletion in 2019 while 42–79% would face it in 2050, confirming the important delay necessary to observe the effect of groundwater consumption on streamflow and the potential magnitude of the phenomenon.^3^ Due to the long response time of aquifers, our groundwater storage DF values likely tend to overestimate, while streamflow DFs underestimate the depletion that would occur at the steady state.

### 3.2 Drivers of Regional Depletion Patterns

Depletion factors for groundwater storage factors span four orders of magnitude, and all other hydrological indicators’ depletion factors span three orders of magnitude across river basins, showing the importance of spatial differentiation (Figure 2 and Table 3). Below, we explain the spatial patterns of the DFs, which reflect the intensity of regional depletion in the water compartments, the type of water use (e.g., irrigation), intercompartment exchanges (e.g., controlled by groundwater heads), and aridity.

High streamflow depletion due to consumption is found not only in arid regions (−1 to −0.5), such as the Mediterranean, East Australia, Central America, and Southern South America, but also in Europe and the Amazon region. Only a few river basins show streamflow increase (5% of the analyzed landmass) in arid warm regions (e.g., Australia, Arabic peninsula), where streamflow is larger with consumption than without consumption. This is possibly because ∼80% groundwater withdrawals for industry and domestic use were returned to streamflow, compensating surface water withdrawals (a similar conclusion was drawn by de Graaf et al.^45^).

Globally, evapotranspiration has increased overall due to water consumption between 1960 and 2000 (see DF values in Figure 2). At the country scale, the analysis of remote sensing data showed that irrigated agriculture has increased evapotranspiration in Brazil, China, Benin, India, Pakistan, Germany, and Thailand, which is consistent with the positive evapotranspiration DF distribution observed in Figure 2.^62^ In addition to the irrigation effect, evapotranspiration increase is also found in regions, where the main consumption drivers are domestic water use, such as in tropical Africa (Congo DF_ET_ = 1.02). Overall, strong evapotranspiration increase is found in arid regions, such as Australia, e.g., in the river basin Murray-Darling, where DF_ET_ = 1.57 is 6 times higher than the global median value. One possible explanation is the very high potential evaporation rates in these regions, which causes return flows from groundwater withdrawals or desalination tend to evaporate rather than return to rivers, aquifers, or soil. In contrast to the above trends, evapotranspiration depletion was found in 17% of the landmass, for example, in Northern Europe (−0.1 to 0), Northern North America (−0.1 to 0), and Malaysia and Indonesia (−0.1 to −1). Even though water consumption can increase the evapotranspiration in a grid cell, other trends can reduce it at the basin scale. In the case of Northern North America and Northern Europe, soil drying (negative DF_SM_) can explain the reduction of evapotranspiration.

Variability in DF_ET_, DF_GWS_, and DF_SM_ relates also to the feedback between groundwater, soil moisture, and evapotranspiration changes, which are driven by groundwater table depth.^61,63^ In regions with a shallow water table, groundwater indirectly supports evapotranspiration via capillary rise (see the map of regions, where capillary rise occurs in Figure S8).^61^ In irrigated crop fields, evapotranspiration increases, and groundwater and soil moisture vary simultaneously because soil moisture is driven by infiltration and capillary rise (e.g., Indus, Niger DF for groundwater, and soil moisture have the same sign, Table 3). Evapotranspiration and soil moisture are not sensitive to groundwater depletion if the groundwater table is already low and capillary rise from the groundwater to the soil is negligible (e.g., Paraná, Sacramento DF for groundwater and soil moisture have opposite signs, Table 3).^63^

We found groundwater storage depletion for regions where groundwater overexploitation has been reported previously, e.g., in the Alluvial River basin of Arizona (−0.5 to −0.1), Mississippi embayment (Mississippi: −0.034), Indo-Gangetic aquifer (Indus: −0.028) (e.g., see^5,64,65^). Groundwater depletion is most severe in regions, where water consumption is high and surface water is scarce, e.g., the Indus river basin. Moreover, severe depletion (<−1) corresponds to regions, where long aquifer response times (>10.000 year) and small recharge rates (e.g., estimated by Cuthbert et al.^61^) can be observed, such as in Australia, Western USA, or in the Arabic peninsula. Positive groundwater depletion factors are found in Northern Europe (>0.01), Eastern China (Yangtze: 0.0085), and North-Eastern Brazil (São Francisco: 0.093), North-Eastern USA (Hudson: 0.07), corresponding to water gains in the aquifer due to consumption. In irrigated regions, groundwater gain relates to the infiltration of water used for irrigation, which can compensate for groundwater withdrawals (e.g., São Francisco, Yangtze), while in other regions where irrigation is not significant, the gain of groundwater may relate to groundwater lateral flows or surface water seepage rather than local consumption.

Depletion factors >1 or <−1, (e.g., Danube and Murray-Darling rivers) correspond to a water gain or loss superior to WC. These changes can be compensated by losses or gains in other compartments of the same river (e.g., surface water storage) or neighbor rivers (through lateral groundwater flow changes).^47^ For instance, evapotranspiration gain in the Murray-Darling river (DF_ET_ = 1.57) is compensated by losses in the other compartments (DF_Q_ + DF_SM_+ DF_GWS_ = −0.92) and gains from other compartments or neighbor basins (1.57–0.92 = −0.65). Similarly, the Danube river loses water to other river basins or in other compartments not mapped by the DFs (sum DFs = −0.97). Therefore, water consumption in the neighbor river basin can influence local depletion. Extreme values for DF_GWS_ and DF_SM_, e.g., in the Zambezi river, the Amazon, and the Congo river, suggest lower accuracy of the GSGM outputs and underestimation of water consumption in these regions.^46^ We analyze the effect of GSGM uncertainty on the DFs in detail in Section 3.5.

### 3.3 Hotspot Regions for Water Depletion

Major hotspots of combined surface and groundwater depletion are revealed when overlaying the maps of negative DFs for groundwater storage and streamflow. These regions include the Amazon, North of Argentina, Central America, Sahel, Eastern Africa, North America, the Mediterranean, Central and Eastern Europe, parts of North-Eastern Russia, the Middle East, Central Asia, Pakistan, Mekong, North China, and Eastern Australia. Most of the hotspot river basins have been reported to be water stressed for 1–4 months annually, except the Amazon, which is not water stressed but is still negatively impacted by consumption.^66^ Therefore, water efficiency improvements and consumption reduction schemes should be implemented as priority in these river basins while keeping in mind social equity, for example, increasing irrigation efficiency and reallocating water to higher water-productivity sectors.^9,67^

Moreover, putting the depletion factors in perspective with the historical change of the water system highlights the specific influence of blue water consumption on its evolution. Our results (Figure 2) and the identified hotspot regions indicate that water consumption contributes to observed streamflow reduction in mid and tropical latitudes and to soil drying in North Africa, Eastern Asia, Europe, and North America, which may lead to irreversible damage to terrestrial and aquatic ecosystems.^68,69^

### 3.4 Surface Water and Groundwater Consumption Effects

The depletion factors include the intertwined effects of surface water and groundwater consumption, but each source of water consumption has a different contribution to freshwater availability change. For instance, surface water withdrawals have a direct effect on streamflow. In contrast, groundwater withdrawals have a direct impact on groundwater storage and an indirect impact on streamflow stemming from the groundwater–surface water connection. The return flows (i.e., the water that is used but not consumed) also influence the final surface and groundwater availability in different ways and depending on the water use. For example, when groundwater is used for industry or domestic uses, return flows go to surface water (Figure S7), while when surface water is used for irrigation, it infiltrates in the soil (Figure S7). This changes the timing of the freshwater resource since surface water compartment residence time is much shorter than groundwater residence time. As a result, both the withdrawal compartment and the return flow compartment influence the duration and the volume of the water availability change, thus the DFs. Given the nonlinearity of the system as exemplified above, disentangling the effect of each water source remains a nontrivial issue out of the scope of this study.

### 3.5 Limitations and Research Needs

In this study, we propose the first step toward the operationalization of the multimedia fate factor framework proposed by Núñez et al., exploring the possibilities offered by state-of-the-art global hydrological model outputs.^18^ While our depletion factors fulfill several requirements discussed in the framework, such as spatial differentiation, global geographical coverage, mechanistic modeling of the exchanges between the compartments, and regional water consumption effects, other aspects need further research.

The adopted water budget approach at the river basin scale does not allow to quantify depletion occurring in a different river basin. Aquifer boundaries do not correspond to river basin boundaries, especially in the 2890 river basins, where groundwater response time is short (<50 years) and lateral groundwater transfers significantly.^47^ In these cases, blue water consumption in one river basin can contribute to freshwater availability change in another neighbor basin due to lateral groundwater transfer. It was however not possible to include capture zones, i.e., the portion of groundwater affected by water consumption, in this study, because the precise location of wells and their pumping flows are unknown at the global scale.^51^ In addition, the aggregation of the hydrological indicators at the river basin scale may be too coarse to highlight local water depletion in large river basins, like the Congo or the Amazon, or smaller aquifer systems, and differences between irrigated and nonirrigated land.^70^ These limitations should be addressed in future studies focusing on sub- or interriver basin capture zones.

Another relevant improvement could be to calculate distinct depletion factors for the effect of surface water withdrawal from groundwater withdrawal, as they have different effects on the water cycle. Our approach was to quantify historical depletion, which results from the intertwined and nonlinear effect of surface and groundwater consumption. Therefore, where both surface and groundwater are consumed, attributing a share of depletion to surface or groundwater consumption was unpractical. As a result, the depletion factors cannot be used to assess whether consuming surface water or groundwater within a river basin causes more depletion. An analytical framework such as the one used by Bierkens et al. may be an approach to explore.^71^

Moreover, DF_ET_ quantifies the evaporation changes induced by blue water consumption but not the related precipitation changes because the GSGM is not coupled with an atmospheric model. The DF_ET_ could be combined with evapotranspiration recycling indicators to estimate the change of precipitation over land induced by blue water consumption.^25,26^ Because of this feedback loop, future studies using GSGMs could consider dynamic precipitation rates rather than observed precipitation data.

Other sources of uncertainty influence the results such as the GSGM and other modeling aspects. Our results are tied to the GSGM outputs uncertainty, which, in turn, reflect the uncertainty in climate forcing and underlying datasets for parametrization.^3,43−46,54,72,73^ The uncertainty is the lowest in regions, where sufficient robust data is available, e.g., USA, Canada, Australia, and Europe. The GSGM performance for streamflow is reported to be lower in the Rocky Mountains, where snow dynamics dominate (as these processes are not well captured in the model), Eastern Europe, and African rivers (in particular the Niger) where groundwater parametrization needs improvements.^46^ It performs insufficiently for total water storage (hence including discharge, soil moisture, and groundwater storage simulations) in the Amazon River, and intertropical rivers in Africa (e.g., Nile, Niger) due to issues with the meteorological forcing data accuracy (e.g., precipitation) and groundwater response time parametrization issues and in high latitude basins (e.g., Yukon River, Iceland) due to deficiencies in modeling ice processes.^46^ In addition, the GSGM performance assessment shows that Malaysia, Japan, Patagonia, the Congo, and the Zambezi regions perform poorly as well.^46^ As a consequence of the GSGM lower performance in these regions, the DFs are more uncertain and should be interpreted accordingly. The list of river basins included in these regions was not possible to establish because no quantitative criteria for judging the GSGM uncertainty was published together with the validation data.^46^ Therefore, such a list should be established by the potential user of the DFs on a case-by-case basis.

Moreover, the dynamic water demand allocation scheme may introduce uncertainty in the DF because the groundwater fractions are underestimated or overestimated compared to observed data, and the extraction of surface or groundwater has different effects.^45^ Domestic and industrial water consumption is underestimated, especially in regions where withdrawals for thermoelectric power plant cooling is significant, such as eastern Europe, France, the UK, Russia, and the eastern USA.^46^ Water consumption by small agricultural water users is also underestimated.^46^ This results in a systematic overestimation of the depletion factors, which partly explains DF values >1 or <−1. Thus, the depletion hotspot regions should be investigated further to confirm the results with local models or field observations. Nevertheless, overall trends and anomalies are well captured by the GSGM; thus, the DFs are suitable for comparative studies across river basins.

The depletion factors were calculated for selected hydrological indicators essential for freshwater-dependent ecosystems and human communities, but not for all of the hydrological variables. Water availability in other compartments of the water cycle may be affected by consumption, such as the surface water storage, precipitation, or lateral groundwater flows inter-river basins. These changes are also modeled in the GSGM but we do not provide DFs for these other compartments because they are less relevant to ecosystems and freshwater resource conservation. Nevertheless, the water balance is closed for each grid cell in the GSGM and by extension in the river basins.^46,3^ Thus, we assume that the depletion factors represent adequately the water balance.

We considered soil moisture and evapotranspiration as hydrological variables (DF numerator) rather than consumptive green water flows (DF denominator) because our focus was on the effect of blue water consumption on the water cycle. Therefore, DF_ET_ captures the blue part of evapotranspiration but it does not capture the blue–green water consumption (soil moisture) induced by land use change.^34,74^ The response of the hydrological cycle to land use change could be quantified using the depletion factor approach, but comparing GSGM outputs for a natural land cover and entropized land cover.^50^

Historical depletion from 1960 to 2000 is not representing steady-state effects of water consumption; hence, it should be primarily used for retrospective assessments. They might be relevant to understand the dynamics of post-2020 freshwater flows and storages, where past consumption practices continue, but periodic depletion factor update is needed to represent adequately the future evolution of the water system, e.g., every decade. In particular, updates would capture changes in surface–groundwater interactions and precipitation/evapotranspiration under climate change.

### 3.6 Potential Applications for Impact Assessment and Water Management

The developed depletion factors show how sensitive key hydrological compartments are to water consumption changes at the river basin level globally. Such new assessment capability can be used in several contexts to support environmental impact assessment and water use management strategies. Depletion factor use should be restricted to areas where the GSGM performance is good, excluding the regions mentioned in Section 3.5.

First, depletion factors can be used to operationalize water sustainability assessment. Several authors proposed to shift from a single freshwater resource planetary boundary to multiple sub-boundaries to preserve key water functions in the global earth system i.e., hydroclimate regulation, terrestrial and aquatic biosphere support, and storage.^29,75^ These sub-boundaries cover the main water compartments—atmospheric water, soil moisture, surface water, groundwater, and frozen water, and are represented by interim indicators (called control variables), namely, evapotranspiration, carbon uptake, streamflow, baseflow, and ice sheet volume, respectively. Except frozen water, our compartments and associated DFs correspond to the sub-boundary scope, providing a tool to tie together water consumption, multicompartment depletion, and potential damage to aquatic and terrestrial ecosystems. Thus, depletion factors may also be useful to convert the safe operating space within each sub-boundary, for example, the minimum streamflow to preserve aquatic ecosystems in a river basin, into sustainable water consumption allowance. Future research could investigate how to connect the depletion factors to each freshwater sub-boundary or even if the selected hydrological indicators in this study could be relevant control variables.

Moreover, the resulting DFs can be implemented in life cycle assessment (LCA), for example, to assess the potential impacts of water consumption on ecosystem quality. LCA is typically used to quantify potential environmental impacts associated with products and services from a life cycle perspective. They can be integrated in life cycle impact assessment methodologies to translate blue water consumption to water resource depletion, human health, and ecosystem damage. For example, the water consumption impact assessment on an aquatic ecosystems currently used in the methodology Recipe2016 builds on the assumption that the consumption of 1 m^3^ of water leads to 1 m^3^ of streamflow reduction in any river basin across the world.^22,76^ Using the DF_Q_ developed in our study instead, the consumption of 1 m^3^ of water would lead to a reduction of streamflow ranging from 0.99 to 0.43 depending on the basin (Table 1) and therefore would lead to more accurate characterization factors for water use. The 1:1 assumption might be more appropriate, where DFs are deemed too uncertain.

The use of models including the DFs in LCA is appropriate for systems where the average water supply mix is a reasonable assumption, e.g., unspecified water origin in the Life Cycle Inventory, and for large-scale systems.^77,78^ Therefore, they can be used for modeling average impacts in LCA because the DFs equal the total depletion divided by the water consumption in the river basin.^14,77,78^ Adopting a historical pragmatic approach to setting the reference state, we adopted the year 1960 as the reference state of the DFs for data availability reasons.^14^ Nonetheless, water consumption rates were estimated to be small in 1960 compared to the 2000 level, the difference is stemming mostly from irrigation increases.^5^ Thus, the reference state could be assumed “pristine” for blue water consumption in regions, where irrigation is the main water use and started after 1960 (e.g., not valid for the USA).

Moreover, the depletion factors could be a useful proxy for potential impacts on freshwater resources in LCA. A previous study framing freshwater resources in LCA suggested using an indicator named potential freshwater depletion, defined as the water consumption beyond the renewability rate for a certain period and expressed in m^3^.^37^ An estimate of long-term availability change of streamflow and groundwater storage is the product of DF_Q_, respectively DF_GWS_, with the water consumption. Therefore, we illustrate how to use the DFs in the LCA context to compare the potential impacts of two consumer products in a fictional case study. A company decides to purchase a new part, and there are two options: part A produced in Europe and part B produced in the USA, both of which require the same water consumption volume for their production of 50 m^3^ (see Table S3). To decide which part they will buy, they compare the potential impacts on freshwater availability (expressed in m^3^ freshwater availability change) of each part from cradle to gate (i.e., material production and manufacturing). The company assumes that the consumption volumes entirely come from the Hudson and the Rhine River basins and that it corresponds to the average supply mix of surface and groundwater. The results indicate that part B has lower potential impacts on freshwater resources (aircraft A: −59.5 m^3^ and B: −45 m^3^) because of the higher potential streamflow depletion in the Rhine River and groundwater storage increase in the Hudson River basin (Table S3). Therefore, the company gives preference toward part B.

Given their limitations, our DFs should be applied carefully according to the applicability domain discussed in this paper. While future studies can tackle these limitations, our CFs represent an improvement compared to state-of-the-art assumptions in water use fate factors in LCIA. Thereof, they can provide important insights to water and sustainability managers by indicating which compartments are more vulnerable to water consumption.

## Supporting Information Available

Supporting Information 1: Description of the GSGM outputs, supplementary methods to depletion factors calculation, including supplementary figures and tables (PDF)
Supporting Information 2: Raster files of the basin delineation, cumulated consumption and consumption 1960–2000, and depletion factors (PDF)
Supporting information 3: Description of the layers of the raster file SI2 (TXT)

## Abbreviations

DF: depletion factor
Q: streamflow
GWS: groundwater storage
SM: soil moisture
ET: evapotranspiration
*D*_Q_, *D*_SM,_*D*_GWS_, *D*_SM_: hydrological indicators for each hydrological variable
DF_Q_, DF_SM,_ DF_GWS_, DF_SM_: depletion factor for each hydrological variable
WC: water consumption
LCA: life cycle assessment
LCIA: life cycle impact assessment
EF: effect factor
FF: fate factor
CF: characterization factor
