# Supplementary material for: Multicompartment Depletion Factors for Water Consumption on a Global Scale
Source: Environ Sci Technol. 2023 Feb 28;57(10):4318–31. doi: 10.1021/acs.est.2c04803 (PMC10019336; doi:10.1021/acs.est.2c04803)
Supplement: Supplementary file 2 — es2c04803_si_003.pdf [file es2c04803_si_003.pdf]

Multi-compartment depletion factors for water consumption on a global scale

Supplementary Information 1

Eleonore Pierrat [a], Martin Dorber [b], Inge de Graaf [c], Alexis Laurent [a], Michael Zwicky Hauschild[a], Martin Rygaard[d], Valerio Barbarossa[e,f]

a Section for Quantitative Sustainability Assessment, Department of Environmental and Resource Engineering, Technical University of Denmark (DTU), 2800 Kgs. Lyngby, Denmark

b Industrial Ecology Programme, Department of Energy and Process Engineering, Norwegian University of Science and Technology (NTNU), Høgskøleringen 5, 7034, Trondheim, Norway

c Water Systems and Global Change Group, Wageningen University & Research, Wageningen, Netherlands

d Water Technology and Processes, Department of Environmental and Resource Engineering, Technical University of Denmark, Bygningstorvet 115, 2800 Kgs. Lyngby, Denmark

e Institute of Environmental Sciences (CML), Leiden University, Leiden, The Netherlands

f PBL Netherlands Environmental Assessment Agency, The Hague, The Netherlands

* Corresponding author email: easpi@dtu.dk

- Number of pages: 24
- Number of tables: 3
- Number of figures: 8

1 Supplementary methods

1.1 GSGM description

In this research, a physically-based global-scale surface water- groundwater model (GSGM) (de Graaf et al. 2019) was used to simulate storages and fluxes of the hydrological system. The model consists of the global hydrology and water-resources model PCR-GLOBWB (van Beek et al. 2011, de Graaf et al. 2014; Sutanudjaja et al. 2018) that is dynamically coupled via groundwater recharge and capillary rise, and by groundwater discharge and river infiltration to a two-layer global groundwater flow model simulating lateral groundwater flows (de Graaf et al. 2017). Both confined and unconfined aquifers are parametrized. The coupled model runs at a daily timestep at 5 arc-minute resolution (approximately 10 x 10 km at the equator). The hydrological model includes a water demand and water-use module that dynamically allocates sectoral water demands from irrigated agriculture, industries, households, and livestock to withdrawal of desalinated water, groundwater or surface water based on the availability of these resources (de Graaf et al. 2014). Return flows of unconsumed withdrawn water flowing back to groundwater or surface water resources, are included in the water availability estimate. The return flows are assumed to happen in the same grid cell as withdrawals. Nevertheless, return flows to surface water can influence downstream surface water availability due to river routing, and return flows to groundwater may influence streamflow downstream through surface water-groundwater interaction that is explicitly included in the model. The GSGM simulated groundwater-surface water interactions dynamically at the global scale, which is a prerequisite for analyzing the effects of groundwater withdrawal on streamflow discharge. The model parameterization is based on the currently best available global-scale datasets, including hydrogeological information essential for the aquifer parameterization (Gleeson et al. 2014, de Graaf et al., 2014, 2017).

We refer to Sutanudjaja et al. (2018) for an extensive description of the PCR-GLOBWB model and its parameterization, to de Graaf et al. (2015, 2017) for a detailed description of the global groundwater model and its parameterization, and to Sutanudjaja et al. (2014) for details on the dynamic coupling between the two models. Model results have been extensively validated against observed river discharges, water table depths, fluctuations, and declines (van Beek et al. 2011; de Graaf et al., 2014, 2015, 2017, 2019; Sutanudjaja et al., 2014).

1.2 Outputs from the GSGM description

The outputs from the GSGM used to calculate depletion factors are presented in Table S1. The outputs were generated for 2 model setups: including human water consumption and dams (named human scenario) and without human interventions (natural setup). The outputs in each scenario are indicated with the subscript human or natural.

Table S1. Outputs of the GSGM used to calculate depletion factors. The outputs were retrieved from de Graaf et al. (2019) and Sutanudjaja et al. (2018).

Output name (symbol)	Unit	Spatial resolution	Time resolution	Description	source
Streamflow (q(t))	m^3/s	5 arcmin	Month 1960-2000	average routed surface water discharge flow	de Graaf et al. 2019

Groundwater depth (d(t))	m/month	5 arcmin	Month 1960-2000	average groundwater depth of the top aquifer layer	de Graaf et al. 2019
Soil moisture up ($sm_{up}(t)$)	m	5 arcmin	year 1960-2004	soil moisture of the top soil 0-30 cm	de Graaf et al. 2019
Soil moisture low ($sm_{low}(t)$)	m	5 arcmin	year 1960-2004	soil moisture of lower soil depth 30-150 cm	de Graaf et al. 2019
evapotranspiration (et(t))	m/month	5 arcmin	month 1960-2004 (natural) month 1960-2000 (human)	total evapotranspiration including bare soil and open water	de Graaf et al. 2019
total withdrawals (ww(t))	m/yr	5 arcmin	annual 1960-2000	annual water withdrawals all sectors	Sutanudjaja et al. 2018
groundwater recharge (gwr(t))	m^3/month	5 arcmin	monthly1960-2000	average groundwater recharge	Sutanudjaja et al. 2018
industry withdrawals ($ww_{ind}(t)$)	m/yr	5 arcmin	year 1960-2000	annual industrial water withdrawals	Sutanudjaja et al. 2018
domestic withdrawals ($ww_{dom}(t)$)	m/yr	5 arcmin	year 1960-2000	annual domestic water withdrawals	Sutanudjaja et al. 2018
water consumption non irrigation ($ww_{non-irri}(t)$)	m/yr	5 arcmin	year 1960-2000	annual net water consumption for domestic and industrial use i.e. not for irrigation	Sutanudjaja et al. 2018
area (A)	m^2	5 arcmin	NA	grid-cell area	Sutanudjaja et al. 2018
aquifer storativity (Sy)	m^3/m^3	5 arcmin	NA	volume of groundwater released from a unit area of aquifer for a unit drawdown of groundwater head	Sutanudjaja et al. 2018
Accumulated surface water flow map $flow_{acc}$	m	5 arcmin	NA	accumulated flow map derived from the digital elevation model	Sutanudjaja et al. 2018
digital elevation model (dem)	m above sea level	5 arcmin	NA	elevation above sea level	Sutanudjaja et al. 2018

1.3 Calculation of hydrological indicators and water consumption at the basin scale

- Net water consumption rate at the basin scale wc(t) in m^3/yr

Annual water consumption rate (wc(t) in m^3/yr) is calculated by using the GSGM outputs for total water withdrawals (ww(t)), groundwater recharge (gwr(t)), non-irrigation water consumption ($wc_{non-irri}$(t)), water withdrawals for industrial use (ww_{ind}(t)), and water withdrawals for domestic use (ww_{dom}(t)) (Table S1).

The annual net water consumption (Equation S1) in one grid cell is defined as the difference between water withdrawals from surface and groundwater (ww in m^3/yr), and the return flows from irrigation (rf_{irri} in m^3/yr Equation S2) and non-irrigation ($rf_{non-irri}$ in m^3/yr; Equation S3Equation S20).

Equation S1

$$wc(\mathrm{t})\left[\frac{m^3}{yr}\right] = ww(\mathrm{t}) - rf_{irri}(\mathrm{t}) - rf_{non-irri}(\mathrm{t})$$

Irrigation return flows (rf_{irri} in m/yr) in one grid cell are derived from groundwater recharge (gwr in m/month) estimated (human impacted run minus natural run) at the monthly timestep, then annual average irrigation return flow is calculated as the arithmetic mean for monthly values.

Equation S2

$$rf_{irri}(\mathrm{t})\left[\frac{m^3}{yr}\right] = (gwr_{human}(\mathrm{t}) - gwr_{natural}(\mathrm{t}))$$

Domestic and industrial return flows in one grid cell ($rf_{non-irri}$ in m^3/yr) are estimated comparing non-irrigation water consumption ($wc_{non-irri}$ in m/yr) and with domestic and industrial withdrawals (ww_{dom}, ww_{ind} in m/yr) following (Equation S3).

Equation S3

$$rf_{non-irri}\,(\mathrm{t})\left[\frac{m^3}{yr}\right] = ww_{dom}(\mathrm{t}) + ww_{ind}(\mathrm{t}) - wc_{non-irri}(\mathrm{t})$$

Then, the basin-scale annual net cumulated water consumption ($WC(t)$ in $m^3.yr^{-1}$) is obtained by summing the grid cell j values over the basin grid cells for each time step t (Equation S4).

Equation S4

$$wc(t)\left[\frac{m^3}{yr}\right] = \sum_{grid\ cell\ k} wc_k(t)$$

- Mean annual streamflow at the basin outlet (q(t) in m^3/yr)

Streamflow at the basin outlet is derived from the GSGM outputs streamflow (q(t)) map for each timestep and flow accumulation map ($flow_{acc}$) (Table S1).

Monthly streamflow at the basin outlet for each timestep (t) (q (t) in m^3/month) is estimated by selecting the grid-cell K of the streamflow map (q_K (t) in m^3/s) corresponding to the basin outlet. Then it is multiplied by the duration of the month (3600.24.365/12 =2.628 10^6 s/month). The outlet of the basin K is the grid cell where the maximum accumulated flow (flow_acc) occurs in the basin (Equation S5, Equation S6). Indices k designate the grid cells in the basin

Equation S5:

$$\mathrm{K} = k\ where\left(\max_k\left(flow_{acc_k}\right)\right)$$

Equation S6:

$$q(\mathrm{t})\left[\frac{m^3}{yr}\right] = q_K(t)\ 2.628\ 10^6$$

We calculate the mean annual streamflow q_{yr}(t) (m^3/yr) as the sum of monthly streamflow over the months of the year (Equation S7).

Equation S7:

$$q_{year}(t)\left[\frac{m^3}{yr}\right] = \sum_{j\ months} q(t_j)$$

For simplicity, we note q(t) the basin scale annual average streamflow (m^3/yr).

- Groundwater head at the basin scale (h(t) in m)

In each grid cell k, the monthly average groundwater head (h(t) in m above sea level) is derived from the groundwater depth (d_k(t), in m) and the elevation (dem, in m above sea level) maps (Equation S8).

Equation S8:

$$h_k(t)[m] = dem_k - d_k(t)$$

The monthly groundwater head at the basin scale h(t) in (m) is the average of grid cell values k included in the basin weighted by the area A_k (m^2) of the grid cell (Equation S9).

Equation S9:

$$h(t)[m] = \frac{1}{\sum_k A_k}\sum_k A_k h_k(t)$$

The annual average groundwater head h_{yr}(t) (m/yr) at the basin scale is the arithmetic mean of the monthly groundwater head (Equation S10):

Equation S10:

$$h_{yr}(t)[m] = \frac{1}{12}\sum_{j\ months} h(t_j)$$

For simplicity, we keep the notation h(t) in (m) to designate the basin scale annual average groundwater head.

The annual groundwater head change at the basin scale dh(t) (in m/yr) is then calculated for each year (t) (Equation S11):

Equation S11:

$$dh(t)\left[\frac{m}{yr}\right] = h(t+1) - h(t)$$

- Groundwater storage depletion at the basin scale (gwd(t) in m^3/yr)

Groundwater storage change is derived from the GSGM output monthly average groundwater head for each time step (t) (d(t) in m above sea level) (Table S1).

Monthly groundwater storage change in grid cell k and month (t) $gwd_k(t)$ (m^3/month) is calculated taking into account grid cell area A_k (m^2) and aquifer storativity Sy_k (m^3/m^3) and monthly average groundwater head $h_k(t)$ (Equation S12).

Equation S12:

$$gwd_k(t)\left[\frac{m^3}{month}\right] = A_k Sy_k\ (h_k(t+1) - h_k(t))/(t+1-t)$$

Basin-scale groundwater depletion gwd(t) (m^3/month) is the sum of the groundwater depletion in the grid cell k in the basin. (Equation S13)

Equation S13:

$$gwd(t)\left[\frac{m^3}{month}\right] = \sum_k A_k Sy_k\ (h_k(t+1) - h_k(t))$$

The annual average groundwater depletion $gwd_{yr}(t)$ (m^3/yr) at the basin scale is the arithmetic mean of the monthly groundwater depletion (Equation S14):

Equation S14:

$$gwd_{yr}(t)\left[\frac{m^3}{yr}\right] = \frac{1}{12}\sum_{j\ months} gwd(t_j)$$

For simplicity, we keep the notation gwd(t) in (m^3/yr) to designate the basin scale annual average groundwater depletion.

- Soil moisture at the basin scale (sm(t) in m^3)

Annual average soil moisture in grid cell k (sm_k in m^3) is derived from the GSGM outputs $sm^{low}_k(t)$ and $sm^{up}_k(t)$ (both in m) and area of the grid cell A_k for all time steps (t) (Table S1, Equation S15).

Equation S15:

$$sm_k(t)[m^3] = A_k(sm_k^{low}(t) + sm_k^{high}(t))$$

Annual soil moisture at the basin scale sm(t) in (m^3) is the sum of grid cell k values $sm_k(t)$ in the basin (Equation S16).

Equation S16:

$$sm_{yr}(t)[m^3] = \sum_k sm_k(t)$$

- Evapotranspiration rate at the basin scale (et(t) in m^3/yr)

Grid cell k evapotranspiration rate ($et_k(t)$ in m/month) for all time steps (t) between 1960 and 2000 is one of the GSGM outputs (Table S1).

Evapotranspiration rate at basin scale (et(t) in m^3/month) is the sum of the evapotranspiration for all grid cells k in the basin, multiplied by the grid cell area Ak (in m^2) (Equation S17).

Equation S17:

$$et(t)\left[\frac{m^3}{month}\right] = \sum_k A_k et_k(t)$$

Annual average evapotranspiration rate is the sum of monthly values j over the year (t) (Equation S18):

Equation S18:

$$et_{year}(t)\left[\frac{m^3}{yr}\right] = \sum_{j\ months} et(t_j)$$

For simplicity, we note et(t) (m^3/yr) the basin scale annual average evapotranspiration.

- 10 years running averages calculation

We applied 10 years running averages to reduce the influence of inter-annual variability on the depletion factors. For each variable v(t) (v(t)={ et(t), q(t), h(t), sm(t)}) in the human and natural scenario, we calculated the 10 years running average for year (t) ($v_{10}(t)$) for the period 1960 to 1990 as the arithmetic mean of annual values for the 10 years subsequent to year (t) (Equation S19).

Equation S19:

$$v_{10}(\mathrm{t}) = \frac{1}{10}\big(v(\mathrm{t}) + \cdots + v(\mathrm{t}+9)\big)$$

1.4 Hydrological balance.

Notations

w(t): annual average total water in the basin (m^3) in year t

sw(t): annual average surface water storage (m^3) in year t in the basin

sm(t): annual average soil moisture storage (m^3) in year t in the basin (calculated with Equation S15, Equation S16)

gws(t): annual average groundwater storage (m^3) in year t in the basin

h(t): annual average groundwater head (m) in year t in the basin (calculated with Equation S8Equation S9 Equation S9, Equation S10)

q(t): annual average streamflow rate at the river mouth (m^3/yr) in year t in the basin (calculated with Equation S5, Equation S6, Equation S7)

et(t): annual average evapotranspiration rate in basin (m^3/yr) in year t in the basin (calculated with Equation S17, Equation S18)

wc(t) : annual average water consumption rate in basin (m^3/yr) in year t in the basin (calculated with Equation S1, Equation S2, Equation S3, Equation S4)

p(t) : annual average precipitation rate in basin (m^3/yr) in year t

lf(t) : annual average net lateral groundwater flows (m^3/yr) in year t (positive if enters the basin, negative if it leaves the basin)

Sy: storativity of the aquifer in the basin (represents confined and unconfined aquifer all together) in (m^3/ m^3)

A: area of the river basin (m^2)

Superscript n: variables in the natural scenario, i.e. without water consumption and dams

Superscript h: variables in the human scenario, i.e. with water consumption and dams

Subscript k: grid cells within a river basin

DF_{SW}: surface water storage depletion factor (m^3/ m^3)

DF_{GWS}: groundwater storage depletion factor (m^3/ m^3)

DF_{SM}: soil moisture depletion factor (m^3/ m^3)

DF_{Q}: streamflow at the river mouth depletion factor (m^3/ m^3)

DF_{ET}: evapotranspiration depletion factor (m^3/ m^3)

D_{SW}: surface water storage change 1960 -2000 (m^3)

D_{GWS}: groundwater storage change 1960 -2000 (m^3)

D_{SM}: soil moisture change 1960 -2000 (m^3)

$D_{Q}(t)$: streamflow annual change defined for each year (t) from 1960 to 2000 (m^3/yr)

$D_{ET}(t)$: evapotranspiration annual change defined for each year (t) from 1960 to 2000 (m^3/yr)

Water balance calculations

The total water volume (expressed in m^3) in the basin during year (t) is the sum of storages volumes (each term expressed in m^3) (Equation S20):

Equation S20

$$w^h(t) = sw^h(t) + gws^h(t) + sm^h(t)$$

Between two consecutive years dt = t+1 – t, the change of total water volume in the basin $dw^h(t)$ is the change of storage rate (expressed in m^3) (Equation S21).

Equation S21

$$dw^h(t) = sw^h(t+1) - sw^h(t) + gws^h(t+1) - gws^h(t) + sm^h(t+1) - sm^h(t)$$

$dw^h(t)$ is also the difference between the input of water and the output of water happening during the time interval dt (each term expressed in m^3) (Equation S22):

Equation S22

$$dw^h(t) = p^h(t)dt + lf^h(t)dt - q^h(t)dt - et^h(t)dt - wc^h(t)dt$$

Thus equalizing previous equations expressing the volume change during 1 year $dw^h(t)$ we obtain Equation S23:

Equation S23

$$\begin{aligned} sw^h(t+1) - sw^h(t) + gws^h(t+1) - gws^h(t) + sm^h(t+1) - sm^h(t) \\ = p^h(t)dt + lf^h(t)dt - q^h(t)dt - et^h(t)dt - wc^h(t)dt \end{aligned}$$

If we write Equation S23 for each year (t) between t0=1960 and t=1999, then sum them up we obtain Equation S24 (each term expressed in cumulated m^3):

Equation S24

$$\begin{aligned} sw^h(2000) - sw^h(1960) + gws^h(2000) - gws^h(1960) + sm^h(2000) - sm^h(1960) \\ = \sum_{1960}^{1999} p^h(t)dt + \sum_{1960}^{1999} lf^h(t)dt - \sum_{1960}^{1999} q^h(t)dt - \sum_{1960}^{1999} et^h(t)dt - \sum_{1960}^{1999} wc(t)dt \end{aligned}$$

The discrete Equation S24 can also be written in terms of continuous integral for any time (t) when dt→0 (Equation S25). Each discrete sum is the numerical estimate of a continuous integral.

Equation S25

$$\begin{aligned} sw^h(2000) - sw^h(1960) + gws^h(2000) - gws^h(1960) + sm^h(2000) - sm^h(1960) \\ = \int_{1960}^{2000} p^h(t)dt + \int_{1960}^{2000} lf^h(t)dt - \int_{1960}^{2000} q^h(t)dt - \int_{1960}^{2000} et^h(t)dt - \int_{1960}^{2000} wc(t)dt \end{aligned}$$

If we divide Equation S24 by $\sum_{1960}^{1999} wc(t)dt$ the cumulated consumption (expressed in m^3) and we substitute dt by its value as follows: $dt = t + 1 - t = 1\ (expressed\ in\ yr)$, we obtain Equation S26 (each term is dimensionless):

Equation S26

$$\frac{sw^h(2000) - sw^h(1960)}{\sum_{1960}^{1999}(wc(t).1)} + \frac{gws^h(2000) - gws^h(1960)}{\sum_{1960}^{1999}(wc(t).1)} + \frac{sm^h(2000) - sm^h(1960)}{\sum_{1960}^{1999}(wc(t).1)}$$

$$= \frac{\sum_{1960}^{1999}(p^h(t).1)}{\sum_{1960}^{1999}(wc(t).1)} + \frac{\sum_{1960}^{1999}(lf^h(t).1)}{\sum_{1960}^{1999}(wc(t).1)} - \frac{\sum_{1960}^{1999}(q^h(t).1)}{\sum_{1960}^{1999}(wc(t).1)} - \frac{\sum_{1960}^{1999}(et^h(t).1)}{\sum_{1960}^{1999}(wc(t).1)} - 1$$

When there is no water consumption in the basin (natural scenario), the water balance is influenced by climate only. Recalculating Equation S24 in the natural scenario (same equation but without $\sum_{1960}^{1999} wc(t)dt$ term) we obtain the water balance change that would have occurred if there were no consumption in the river basin. Dividing recalculated Equation S24 in the natural scenario by $\sum_{1960}^{1999} wc(t)dt$, subtracting the result to Equation S26. And replacing dt by its value (1 yr), we obtain Equation S27 (each term is dimensionless). Equation S27 represents the effect of water consumption only on the water balance.

Equation S27

$$\frac{\left(sw^h(2000) - sw^h(1960)\right) - \left(sw^n(2000) - sw^n(1960)\right)}{\sum_{1960}^{1999}(wc(t).1)} + \frac{\left(gws^h(2000) - gws^h(1960)\right) - \left(gws^n(2000) - gws^n(1960)\right)}{\sum_{1960}^{1999}(wc(t).1)} + \frac{\left(sm^h(2000) - sm^h(1960)\right) - \left(sm^n(2000) - sm^n(1960)\right)}{\sum_{1960}^{1999}(wc(t).1)}$$

$$= \frac{\sum_{1960}^{1999}\left(\left(p^h(t) - p^n(t)\right).1\right)}{\sum_{1960}^{1999}(wc(t).1)} + \frac{\sum_{1960}^{1999}\left(\left(lf^h - lf^n(t)\right).1\right)}{\sum_{1960}^{1999}(wc(t).1)} - \frac{\sum_{1960}^{1999}\left(\left(q^h(t) - q^n(t)\right).1\right)}{\sum_{1960}^{1999}(wc(t).1)} - \frac{\sum_{1960}^{1999}\left(\left(et^h(t) - et^n(t)\right).1\right)}{\sum_{1960}^{1999}(wc(t).1)} - 1$$

Equation S27 can also be written in terms of continuous integral when dt→0. We obtain Equation S28.

Equation S28

$$\frac{\left(sw^h(2000) - sw^h(1960)\right) - \left(sw^n(2000) - sw^n(1960)\right)}{\int_{1960}^{2000} wc(t)dt} + \frac{\left(gws^h(2000) - gws^h(1960)\right) - \left(gws^n(2000) - gws^n(1960)\right)}{\int_{1960}^{2000} wc(t)dt} + \frac{\left(sm^h(2000) - sm^h(1960)\right) - \left(sm^n(2000) - sm^n(1960)\right)}{\int_{1960}^{2000} wc(t)dt}$$

$$= \frac{\int_{1960}^{2000}\left(p^h(t) - p^n(t)\right)dt}{\int_{1960}^{2000} wc(t)dt} + \frac{\int_{1960}^{2000}\left(lf^h - lf^n(t)\right)dt}{\int_{1960}^{2000} wc(t)dt} - \frac{\int_{1960}^{2000}\left(q^h(t) - q^n(t)\right)dt}{\int_{1960}^{2000} wc(t)dt} - \frac{\int_{1960}^{2000}\left(et^h(t) - et^n(t)\right)dt}{\int_{1960}^{2000} wc(t)dt} - 1$$

1.5 Depletion factors definition.

We define the depletion factors for selected terms of the Equation S28 that have high ecological relevance (Q, ET, GWS, SM). Depletion factors reflect the influence of water consumption on the water cycle only. The depletion factors numerical values are obtained from Equation S27.

We define WC the cumulated water consumption from 1960 to 2000 in a river basin (expressed in m^3), it can be estimated based on discrete time series of annual water consumption rate wc(t) (expressed in m^3/yr) and the time interval dt=1(expressed in yr) as follows (Equation S29):

Equation S29

$$WC = \int_{1960}^{2000} wc(t)dt \approx \sum_{1960}^{1999}(wc(t).1)$$

We define the groundwater storage depletion factor DF_{GWS} (expressed in m^3/m^3) (Equation S30):

Equation S30

$$DF_{GWS} = \frac{\left(gws^h(2000) - gws^h(1960)\right) - \left(gws^n(2000) - gws^n(1960)\right)}{WC}$$

By definition groundwater storage change from 1960 to 2000 is equal to the groundwater depletion 1960-2000 calculated with (Equation S12, Equation S13, Equation S14). Therefore we obtain Equation S31:

Equation S31

$$DF_{GWS} = \frac{\sum_k\left(A_k\, Sy_k\left(\left(h_k^h(2000) - h_k^h(1960)\right) - \left(h_k^n(2000) - h_k^n(1960)\right)\right)\right)}{WC}$$

We define the soil moisture depletion factor DF_{SM} (expressed in m^3/m^3) (Equation S32).

Equation S32

$$DF_{SM} = \frac{\left(sm^h(2000) - sm^h(1960)\right) - \left(sm^n(2000) - sm^n(1960)\right)}{WC}$$

We define the streamflow depletion factor DF_Q (expressed in m^3/m^3) as the cumulated change of discharge to the ocean and we estimate its value using discrete time series of annual streamflow at the river mouth (Equation S33). The numerator of equation 33 $\int_{1960}^{2000}\left(q^h(t) - q^n(t)\right)dt$ represents the area under the curve $q^h(t) - q^n(t)$ between for each instant (t) from 1960 to 2000. However we do not know the value of $q^h(t)$ and $q^n(t)$ for every instant, we know instead the values for every year (t) from 1960 to 2000. We approximate the integral using the discrete annual

time-series by calculating the sum of the small rectangles of area $\left(q^h(t) - q^n(t)\right).1$ for each year (t) from 1960 to 1999.

Equation S33

$$DF_Q = \frac{\int_{1960}^{2000}\left(q^h(t) - q^n(t)\right)dt}{WC} \approx \frac{\sum_{1960}^{1999}\left(\left(q^h(t) - q^n(t)\right).1\right)}{\sum_{1960}^{1999}(wc(t).1)}$$

Similarly we define the evapotranspiration depletion factor DF_{ET} (expressed in m^3/m^3) as the cumulated change of evapotranspiration and we estimate its value using discrete time series of annual evapotranspiration rate (Equation S34):

Equation S34

$$DF_{ET} = \frac{\int_{1960}^{2000}\left(et^h(t) - et^n(t)\right)dt}{\int_{1960}^{2000} wc(t)dt} \approx \frac{\sum_{1960}^{1999}\left(\left(et^h(t) - et^n(t)\right).1\right)}{\sum_{1960}^{1999}(wc(t).1)}$$

We smooth internal variability of q(t), et(t), h(t), wc(t), and sm(t) by calculating 10 years moving averages for each year between 1960 and 1990 so that depletion factors are not influenced by the inter-annual variability but reflect overall trends. We maintain the same notation for each variable for the sake of readability. For example, q(1967) represents the annual average streamflow at the river mouth between 1967 and 1976. As a result, the hydrological indicators are defined and related to the depletion factors as in Table S2.

Table S2: Hydrological indicator definition and calculation details

Compartment	Hydrological variable	Unit	Description of the hydrological indicator	Depletion factors
Surface water	Streamflow Q(t)	$m^3\ yr^{-1}$	Change of routed surface water discharge at the outlet of the basin between the 1960's and 1990's. $D_Q(t) = \left(q^h(t) - q^n(t)\right)$ where: $q^h(t)$ are the average annual streamflow at the river outlet during the decade (t, t+9) with water consumption. $q^n(t)$ is similar to $q^h(t)$ but calculated in the natural scenario (no water consumption). They are defined for (t) between 1960 and 1990 thus representing the period 1960-2000.	$DF_Q = \frac{\int_{t=1960}^{2000} D_Q(t).dt}{WC} \approx \frac{\sum_{t=1960}^{1990}\left(D_Q(t).1\right)}{\sum_{t=1960}^{1990}(wc(t).1)}$
Groundwater	Groundwater storage cumulated change	m^3	Change of groundwater storage, including confined and unconfined aquifer, between the 1960's and 1990's.	$DF_{GWS} \approx \frac{D_{GWS}}{\sum_{t=1960}^{1999}(wc(t).1)}$

	(D_{GWS})		$D_{GWS} = A\, Sy\left(\left(h^h(1990) - h^h(1960)\right) - \left(h^n(1990) - h^n(1960)\right)\right)$<br><br>where: A, Sy, and h(t) are the area (m^2), aquifer storativity (m^3m^{-3}), and the annual average groundwater head (m) in the basin.<br><br>Therefore, cumulated groundwater storage change is the difference between average groundwater storage volume in the 1990's and 1960's.	
Soil	Soil moisture cumulated change<br><br>(D_{SM})	m^3	Change of soil moisture volume over the top 1.5 m of soil depth between the 1960's and 1990's.<br><br>$D_{SM} = \left(sm^h(1990) - sm^h(1960)\right) - \left(sm^n(1990) - sm^n(1960)\right)$<br><br>where : $sm^h(1990)$ and $sm^h(1960)$ are the annual average soil moisture (m^3) in the decades of 1990's and 1960's in the human scenario (with water consumption). $sm^n(1990)$ and $sm^n(1960)$ are similar to $sm^h(1990)$ and $sm^h(1960)$ but calculated in the natural scenario (no water consumption).	$DF_{SM} \approx \frac{D_{SM}}{\sum_{t=1960}^{1999}(wc(t).1)}$
Atmosphere	Evapotranspiration rate<br><br>(ET(t))	m^3yr^{-1}	Total annual evapotranspiration rate change from vegetation, bare soil, and open water between the 1960's and the 1990's.<br><br>$D_{ET}(t) = \left(et^h(t) - et^n(t)\right)$<br><br>where: $et^h(t)$ is the average annual evapotranspiration rates (m^3yr^{-1}) during the decade (t, t+9) with water consumption. $et^h(t)$ is similar to $et^h(t)$ but calculated in the natural scenario (no water consumption). They are defined for (t) between 1960 and 1990 thus representing the period 1960-2000.	$DF_{ET} = \frac{\int_{t=1960}^{2000} D_{ET}(t).dt}{WC} \approx \frac{\sum_{t=1960}^{1990}(D_{ET}(t).1)}{\sum_{t=1960}^{1990}(wc(t).1)}$

Rearranging Equation S27 and substituting by the depletion factors definition we obtain Equation S35:

Equation S35

$$DF_{GWS} + DF_{SM} + DF_Q + DF_{ET} = \frac{\sum_{1960}^{1999}\left(\left(p^h(t) - p^n(t)\right).1\right)}{\sum_{1960}^{1999}(wc(t).1)} + \frac{\sum_{1960}^{1999}\left(\left(lf^h - lf^n(t)\right).1\right)}{\sum_{1960}^{1999}(wc(t).1)} - \frac{\left(sw^h(2000) - sw^h(1960)\right) - \left(sw^n(2000) - sw^n(1960)\right)}{\sum_{1960}^{1999}(wc(t).1)} - 1$$

We define the term R (dimensionless) is the residual influence of water consumption on other parts (like precipitation, lateral groundwater flows, and surface water storage) of the water balance (Equation S36) in the river basin.

Equation S36

$$R = \frac{\sum_{1960}^{1999}\left(\left(p^h(t)-p^n(t)\right).1\right)}{\sum_{1960}^{1999}(wc(t).1)} + \frac{\sum_{1960}^{1999}\left(\left(lf^h-lf^n(t)\right).1\right)}{\sum_{1960}^{1999}(wc(t).1)} - \frac{\left(sw^h(2000)-sw^h(1960)\right)-\left(sw^n(2000)-sw^n(1960)\right)}{\sum_{1960}^{1999}(wc(t).1)}$$

We obtain finally Equation S37 by substituting the expression of R (Equation S35) in Equation S36:

Equation S37

$$DF_{GWS} + DF_{SM} + DF_Q + DF_{ET} = R - 1$$

2 Case study results

Table S3. Results of the cradle-to-gate impacts assessment of the illustrative case study

	**aircraft A**	**aircraft B**
**total water consumption (m^3)**	50	50
**river basin**	Rhine	Hudson
**DF_Q**	-1.03	-0.97
**DF_{GWS}**	-0.16	0.07
**Potential impact on freshwater resources (m^3)**	surface water: -1.03·50 = -51.5<br>groundwater:-0.16·50 = -8<br>total: (-1.03-0.16)·50 = -59.5	surface water: -0.97·50 = -48.5<br>groundwater: 0.07·50 = 3.5<br>total: (-0.97+0.07)·50 = -45

3 Supplementary figures

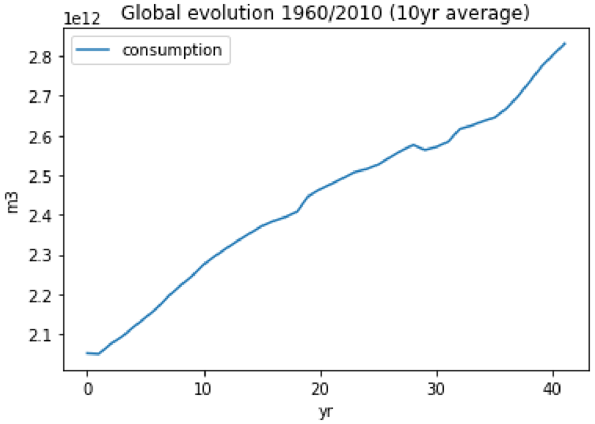

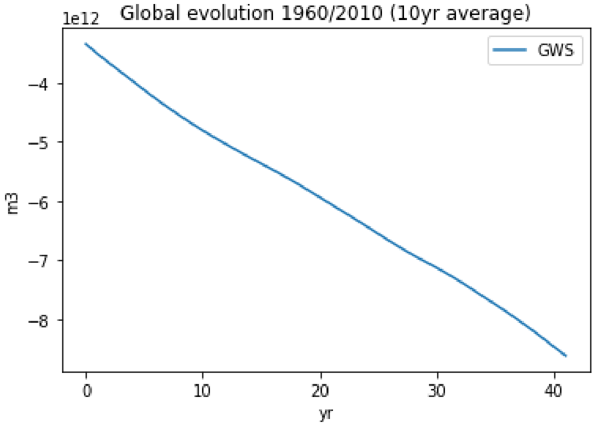

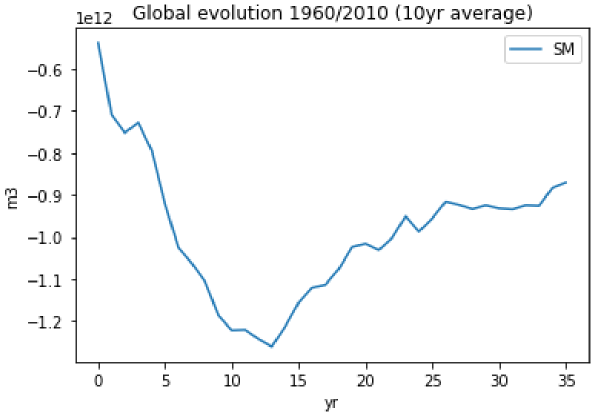

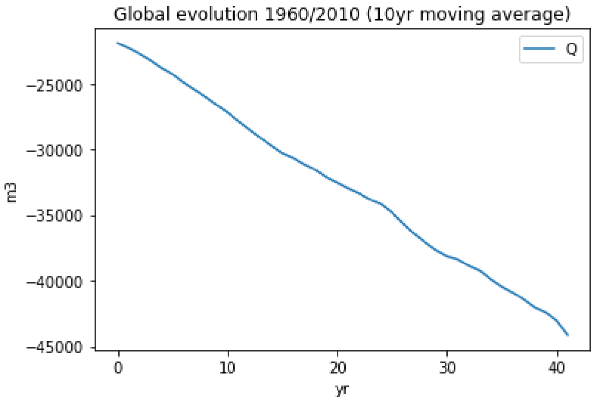

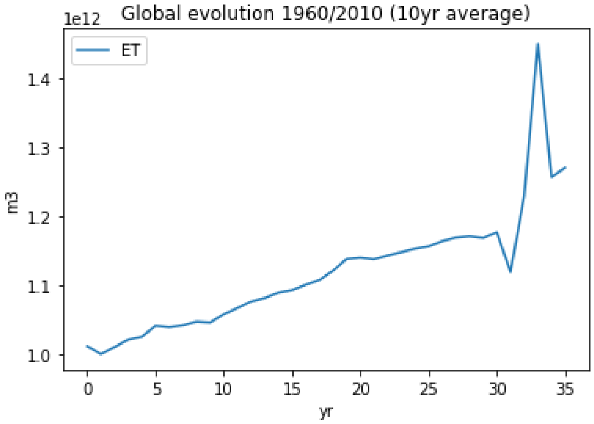

Figure S1. Global change for streamflow, groundwater storage, soil moisture, evapotranspiration and groundwater head drawdown rate from 1960 (year 0) to 2010 (year 40). Change is presented as the difference between the GSGM outputs in the human and natural scenarios and 10 years running averages were applied. Because of the evaporation instability after 2000, we chose to restrict the period over which the depletion factors were calculated from 1960 to 2000 (instead of 2010).

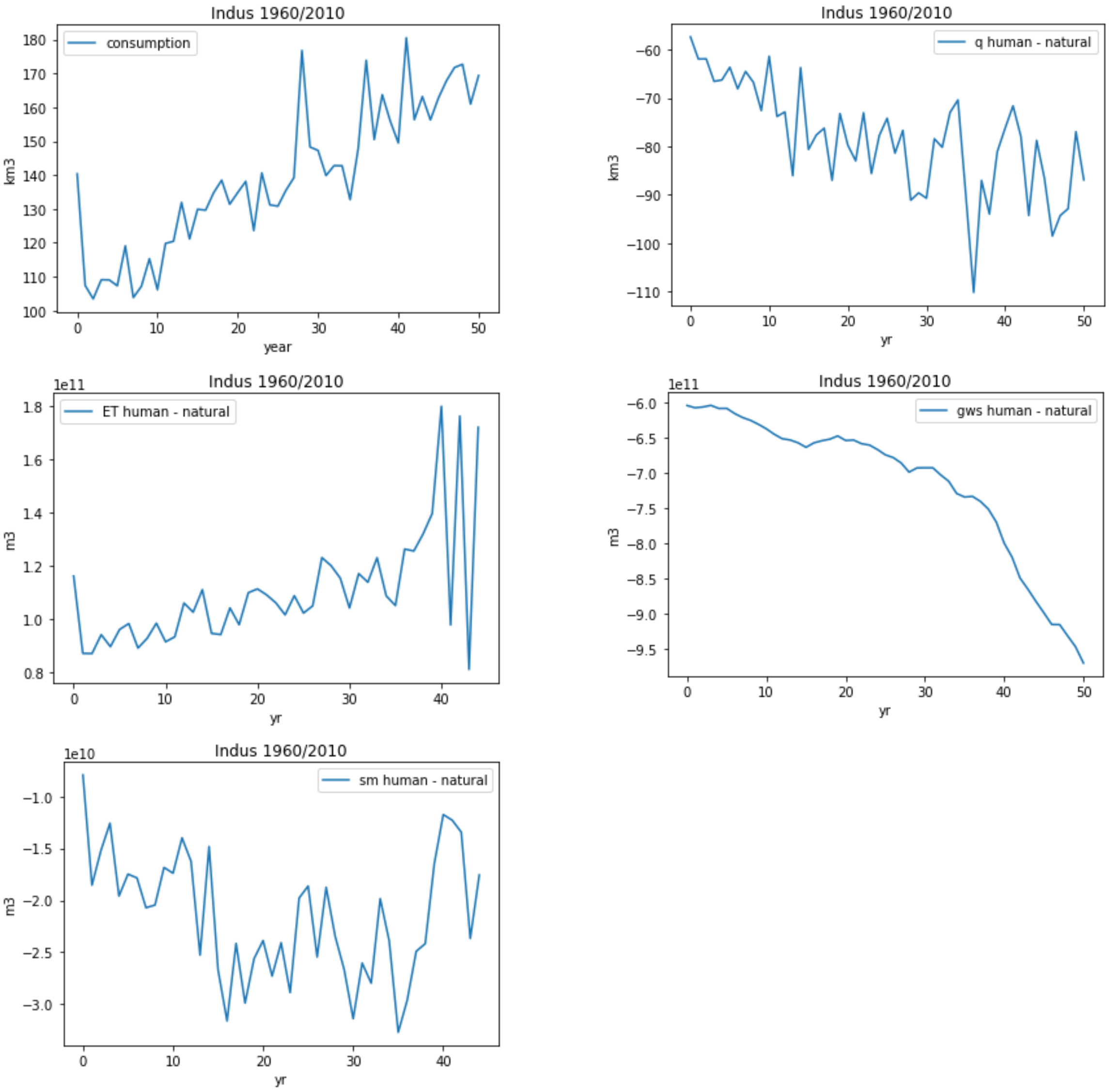

Figure S2. Annual change of the hydrological variables water consumption (consumption), discharge (q human - natural), groundwater storage (gws human - natural), soil moisture (sm human - natural), evapotranspiration (et human - natural) in the Indus basin from 1960 to 2010. Change is presented as the difference between the GSGM outputs in the human and natural scenarios. No smoothing was applied.

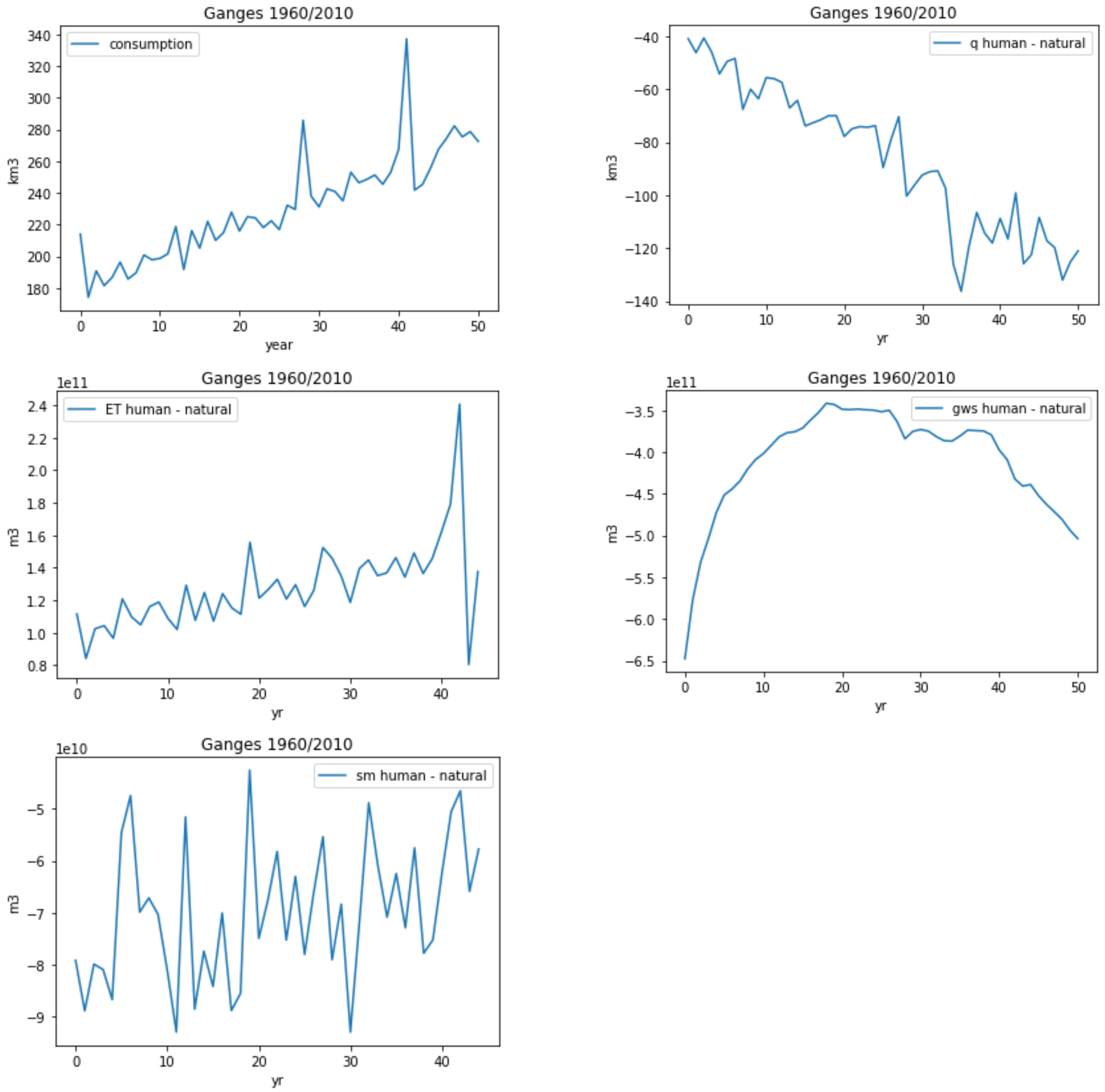

Figure S3: Annual change of the hydrological variables water consumption (consumption), discharge (q human - natural), groundwater storage (gws human - natural), soil moisture (sm human - natural), evapotranspiration (et human - natural) in the Ganges basin from 1960 to 2010. Change is presented as the difference between the GSGM outputs in the human and natural scenarios. No smoothing was applied.

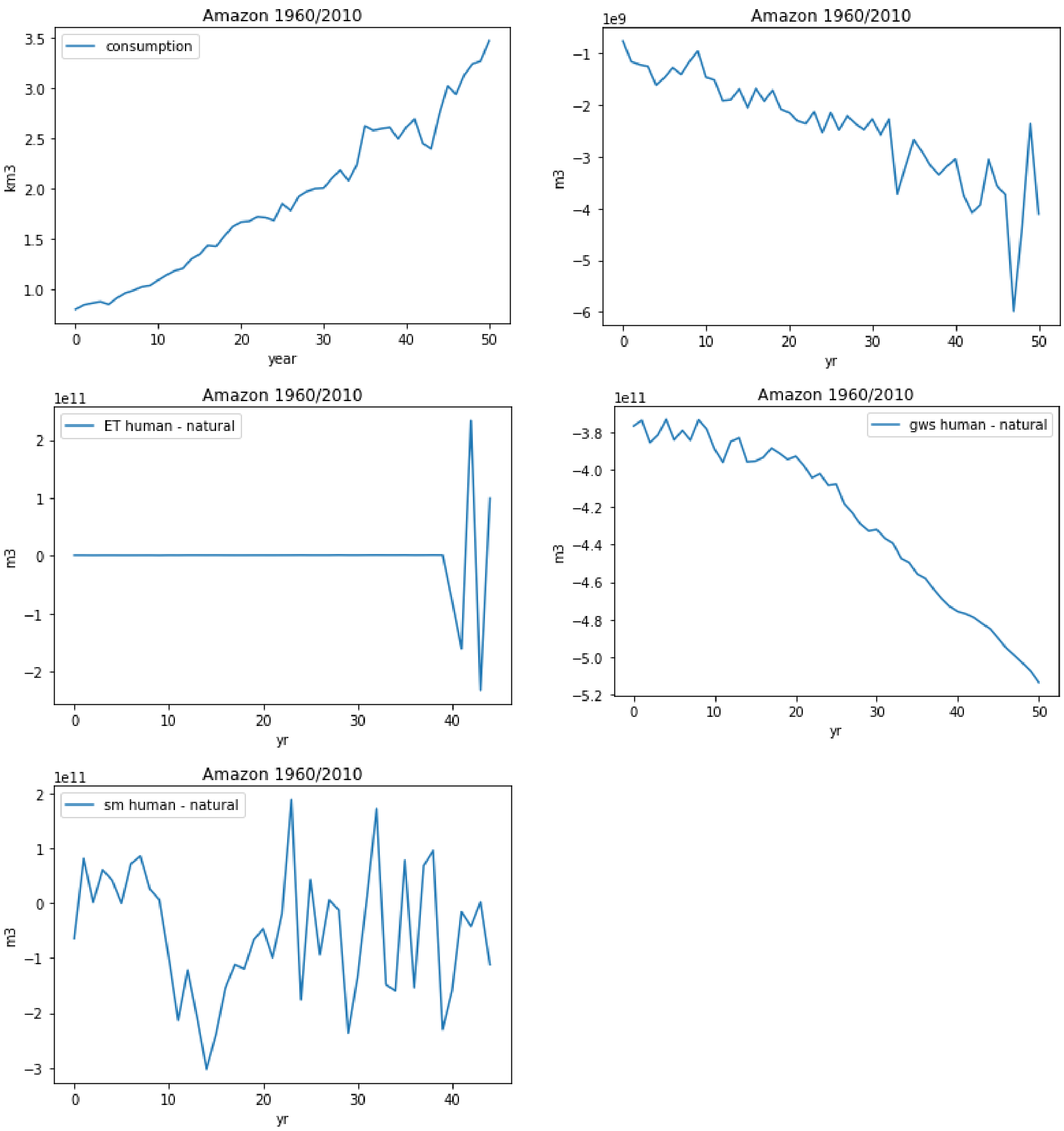

Figure S4 Annual change of the hydrological variables water consumption (consumption), discharge (q human - natural), groundwater storage (gws human - natural), soil moisture (sm human - natural), evapotranspiration (et human - natural) in the Amazon basin from 1960 to 2010. Change is presented as the difference between the GSGM outputs in the human and natural scenarios. No smoothing was applied.

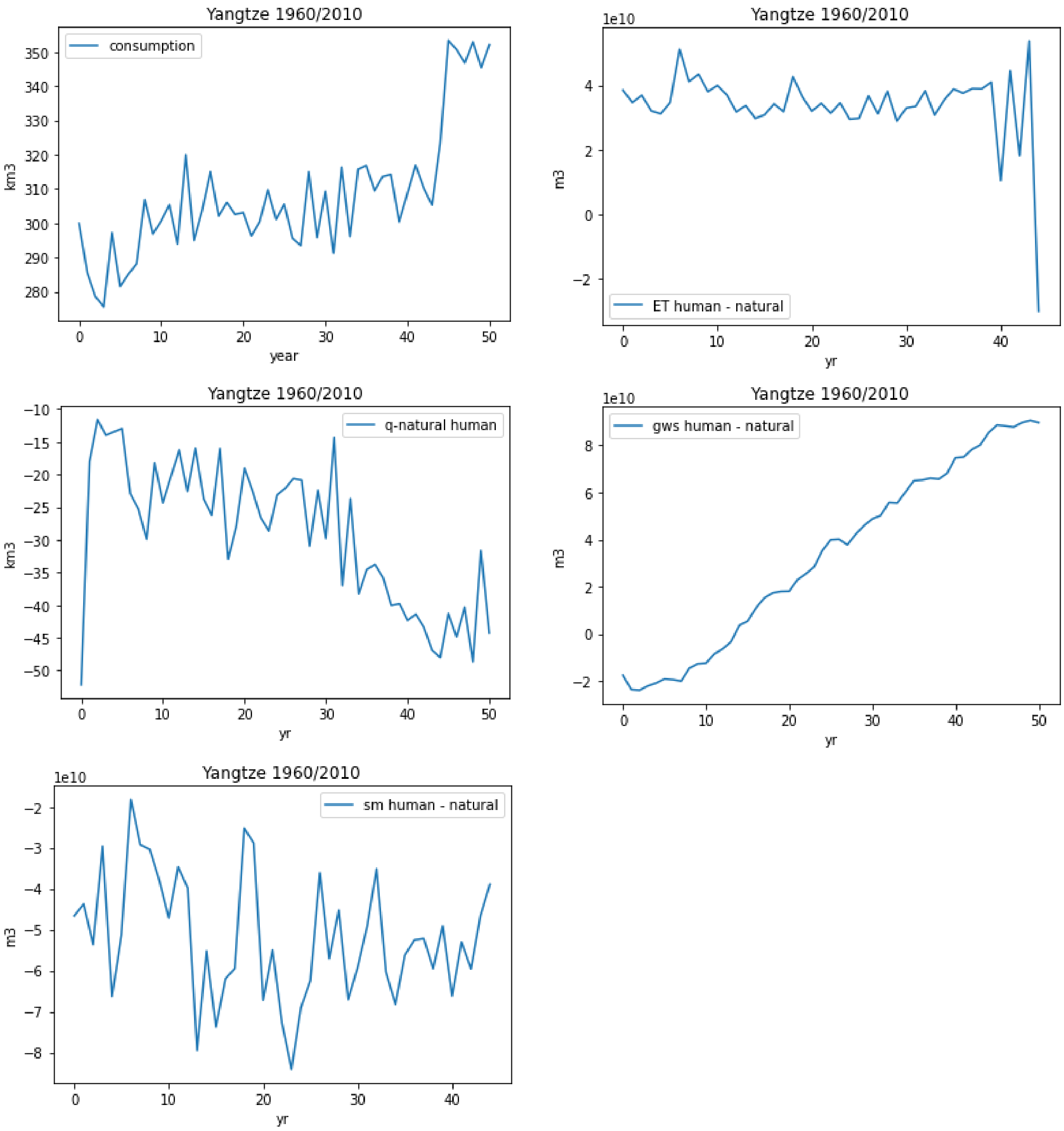

Figure S5 Annual change of the hydrological variables water consumption (consumption), discharge (q human - natural), groundwater storage (gws human - natural), soil moisture (sm human - natural), evapotranspiration (et human - natural) in the Yangtze basin from 1960 to 2010. Change is presented as the difference between the GSGM outputs in the human and natural scenarios. No smoothing was applied.

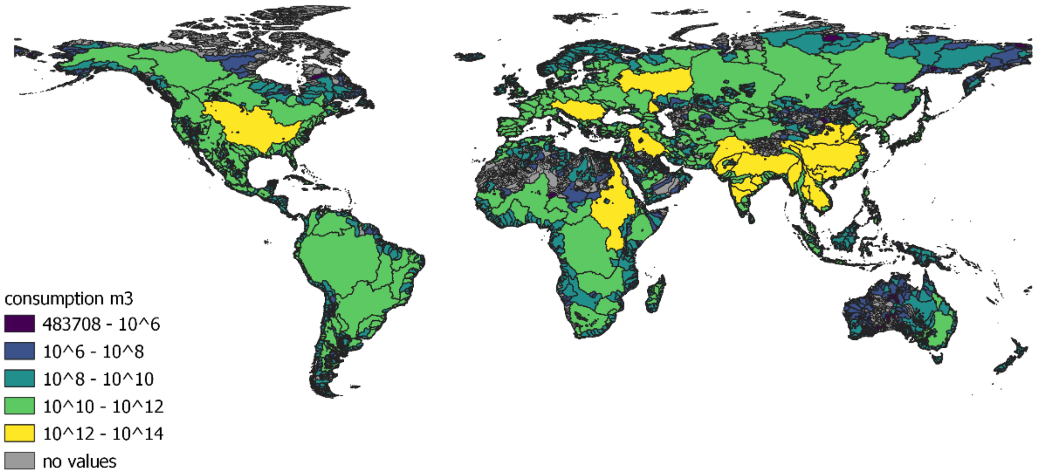

Figure S6. Cumulative net water consumption in river basins 1960-2000 (in m^3). No smoothing was applied.

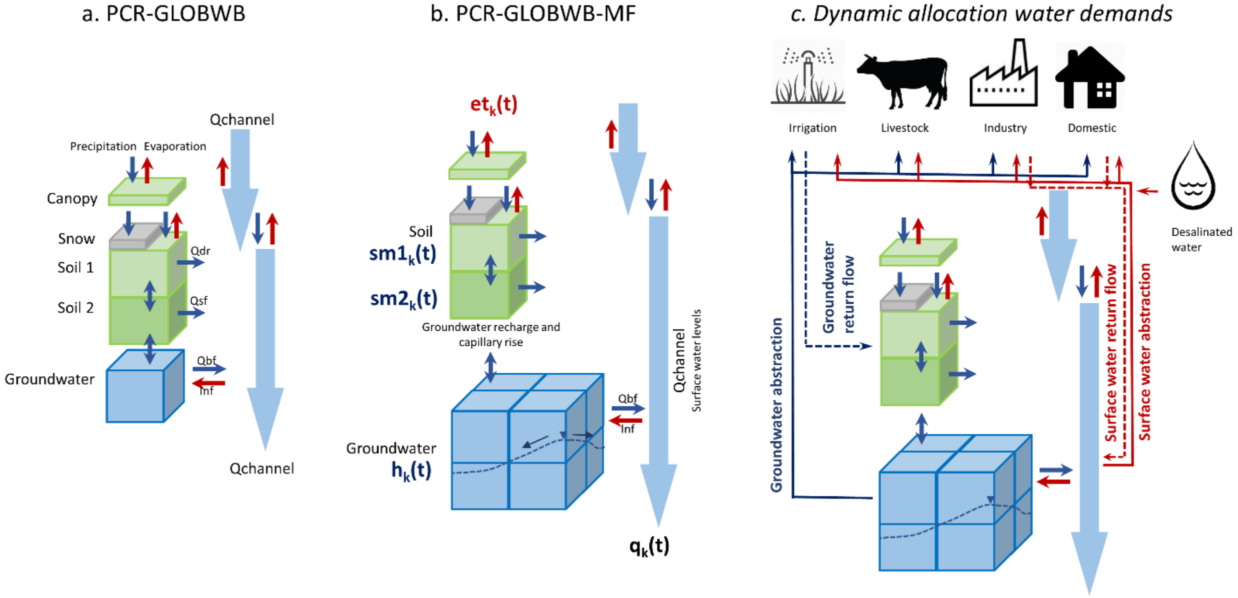

Figure S7: Schematic overview of (a) PCR-GLOBWB, (b) PCR-GLOBWB-MF and (c) dynamic allocation demand for each grid cell and its modelled states and fluxes (adapted from de Graaf and Stahl ([2]) and de Graaf et al. ([3,4])). In the GSGM (Figure S7b), the linear groundwater model of PCR-GLOBWB (Figure S7a) is replaced by the groundwater model Modflow and it includes the water withdrawals and return flows from the water demand and water use module (Figure S7c). The main flows are $sm1_k(t)$, $sm2_k(t)$ (soil moisture storage), $h_k(t)$ (groundwater head), $q_k(t)$ (streamflow), Qdr (surface run-off – from rainfall and snowmelt), Qsf (interflow or stormflow), Qbf (baseflow or groundwater discharge), and Inf (riverbed infiltration from to groundwater). In the water demand and water use module (Figure S7c), the thin red lines indicate surface water withdrawal, the thin blue lines groundwater abstraction, the thin red dashed lines return flows from surface water use, and the thin dashed blue lines return flows from groundwater use surface. Water consumption is equal all withdrawals minus return flows. The standard model input and outputs in bold, dark blue and dark red fronts are extracted from the GSGM and the water demand and water use module to calculate the depletion factors.

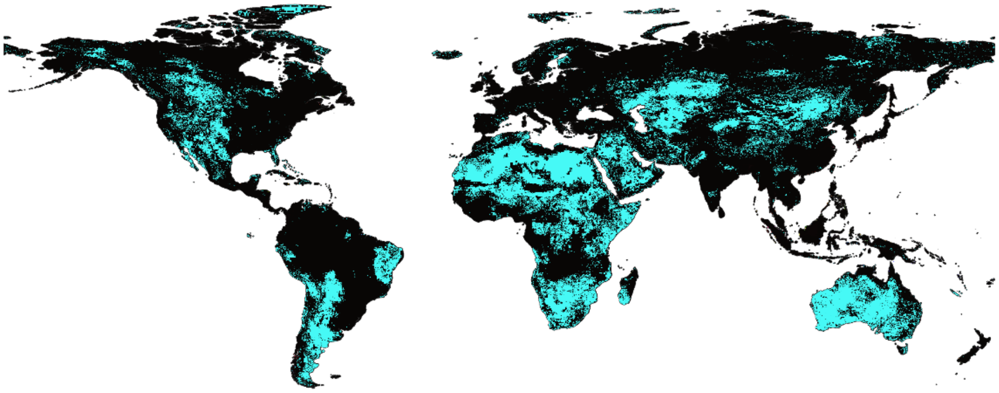

Figure S8: Capillary rise occurrence in 2000 (in blue). The capillary rise occurs when the annual average groundwater recharge is negative.

4 Supplementary References

De Graaf, I. E. M.; van Beek, L. P. H.; Wada, Y.; Bierkens, M. F. P. Dynamic Attribution of Global Water Demand to Surface Water and Groundwater Resources: Effects of Abstractions and Return Flows on River Discharges. *Adv. Water Resour.* 2014, *64*, 21–33. https://doi.org/10.1016/j.advwatres.2013.12.002.

De Graaf, I. E. M.; Sutanudjaja, E. H.; Van Beek, L. P. H.; Bierkens, M. F. P. A High-Resolution Global-Scale Groundwater Model. *Hydrol. Earth Syst. Sci.* 2015, *19* (2), 823–837. https://doi.org/10.5194/hess-19-823-2015.

de Graaf, I. E. M.; van Beek, R. L. P. H.; Gleeson, T.; Moosdorf, N.; Schmitz, O.; Sutanudjaja, E. H.; Bierkens, M. F. P. A Global-Scale Two-Layer Transient Groundwater Model: Development and Application to Groundwater Change. *Adv. Water Resour.* 2017, *102*, 53–67. https://doi.org/10.1016/j.advwatres.2017.01.011.

de Graaf, I. E. M.; Gleeson, T.; (Rens) van Beek, L. P. H.; Sutanudjaja, E. H.; Bierkens, M. F. P. Environmental Flow Limits to Global Groundwater Pumping. *Nature* 2019, *574* (7776), 90–94. https://doi.org/10.1038/s41586-019-1594-4.

Gleeson Tom, Nils Moosdorf, Jens Hartmann, L. P. H. van B. Geophysical Research Letters. *Geophys. Prospect.* 2014, No. April, 6298–6305. https://doi.org/10.1002/2014GL059856.Received.

Sutanudjaja, E. H., van Beek, L. P. H., De Jong, S. M., Van Geer, F. C. & Bierkens, M. F. P. Calibrating a large-extent high-resolution coupled groundwater–land surface model using soil moisture and discharge data. *Wat. Resour. Res*. 2014, 50, 687–705.

Sutanudjaja, E. H.; Van Beek, R.; Wanders, N.; Wada, Y.; Bosmans, J. H. C.; Drost, N.; Van Der Ent, R. J.; De Graaf, I. E. M.; Hoch, J. M.; De Jong, K.; Karssenberg, D.; López López, P.; Peßenteiner, S.; Schmitz, O.; Straatsma, M. W.; Vannametee, E.; Wisser, D.; Bierkens, M. F. P. PCR-GLOBWB 2: A 5 Arcmin Global Hydrological and Water Resources Model. *Geosci. Model Dev.* 2018, *11* (6), 2429–2453. https://doi.org/10.5194/gmd-11-2429-2018.

Van Beek, L. P. H.; Eikelboom, T.; Van Vliet, M. T. H.; Bierkens, M. F. P. A Physically Based Model of Global Freshwater Surface Temperature. *Water Resour. Res.* 2012, *48* (9). https://doi.org/10.1029/2012WR011819.
